# Supplementary material for: Buffer optimization of siRNA-lipid nanoparticles mitigates lipid oxidation and RNA-lipid adduct formation
Source: Nat Commun. 2025 Sep 25;16:8380. doi: 10.1038/s41467-025-63651-4 (PMC12462517; doi:10.1038/s41467-025-63651-4)
Supplement: Supplementary file 1 — Supplementary Information [file 41467_2025_63651_MOESM1_ESM.pdf]

Supplementary Information

for

Buffer Optimization of siRNA-Lipid Nanoparticles Mitigates Lipid Oxidation and RNA-lipid Adduct Formation

Daniel A. Estabrook[1*], Lihua Huang[2], Olivia R. Lucchese[1], Dylan J. Charland[1], Zhao Yu[2], Fareed Bhasha Sayyed[3], Jonas Y. Buser[2], Younghoon Oh[1], Xingyan (Astra) Liu[1], Harmon A. Johnson[1], Kenneth G. Rodriguez[1], Noah A. Wambolt[4], Sonia A. Corba[4], Geoffrey T. Nash[2], Dennis Yang[2], Tingting Wang[2]

Affiliations:

[1]*Lilly Seaport Innovation Center, 15 Necco St, Boston, Massachusetts 02210, USA*

[2]*Eli Lilly and Company, 1200 West Morris St., Indianapolis, Indiana 46225, USA*

[3]*Eli Lilly Services India Pvt Ltd., Bengaluru 560103, India*

[4]*Eurofins Lancaster Laboratories Professional Scientific Services, LLC, Lancaster, Pennsylvania 17601, USA*

*email: estabrook_daniel@lilly.com

Table of Contents

Abbreviations……………………………………………………….……………………………3

Supplementary Figures and Experimental Procedures………………………………………….4

Supplementary Tables…………………...…………………………………………….……….….28

Abbreviations:

LNP – lipid nanoparticle

MC3 – DLin-MC3-DMA

DSPC – distearoylphosphatidylcholine

TFF – tangential flow filtration

TMP – trans-membrane pressure

RT – room temperature, ~22–25 °C

DLS – dynamic light scattering

MFI – micro-flow imaging

LC/MS – liquid chromatography mass spectrometry

PDI – polydispersity index

EE% - encapsulation efficiency percentage

PBS – phosphate buffered saline

RDF – radial distribution functions

siRNA – short interfering ribonucleic acid

mRNA – messenger ribonucleic acid

NMR – nuclear magnetic resonance

EtOH – ethanol

NaOH – sodium hydroxide

Supplementary figures and experimental procedures

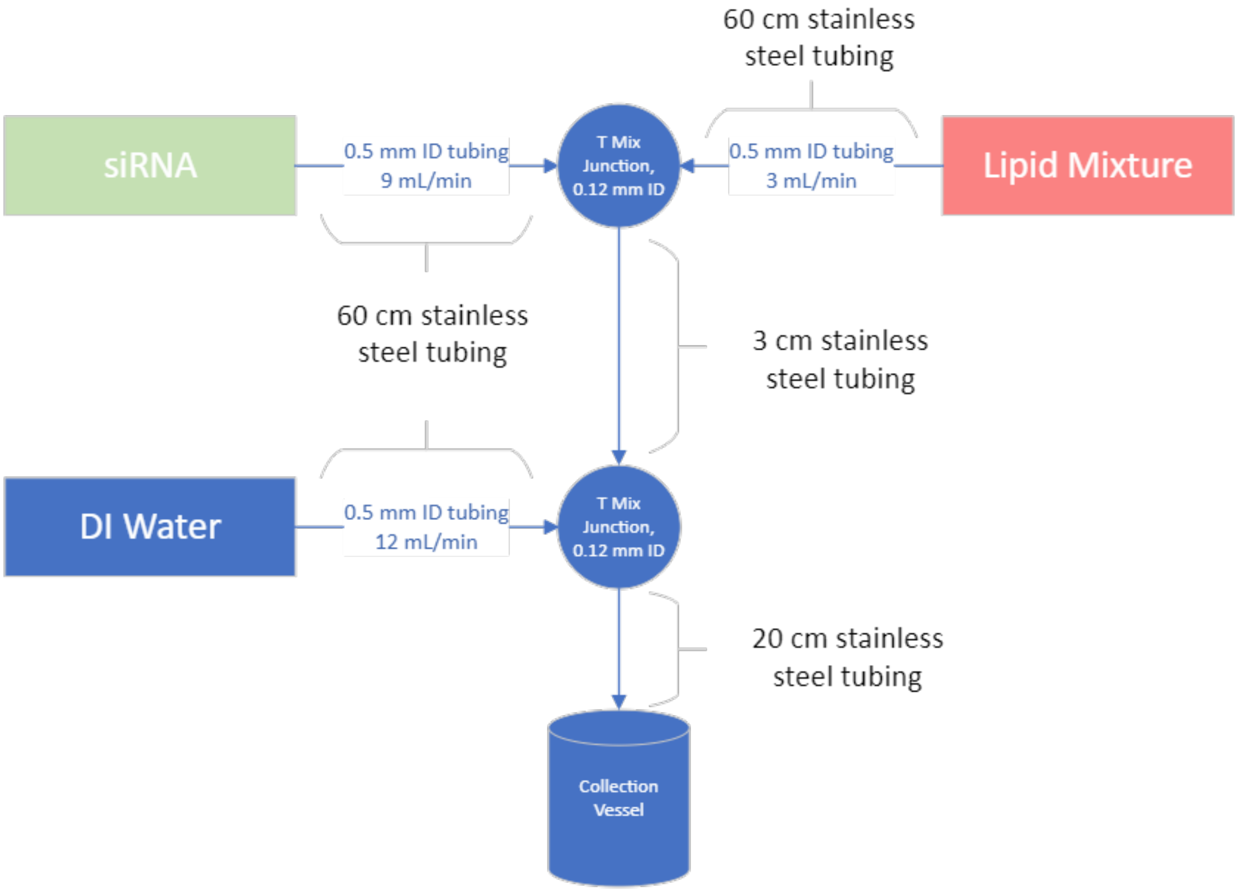

Supplementary Figure 1. Schematic of syringe pump mixing configuration used for RNA-LNP generation.

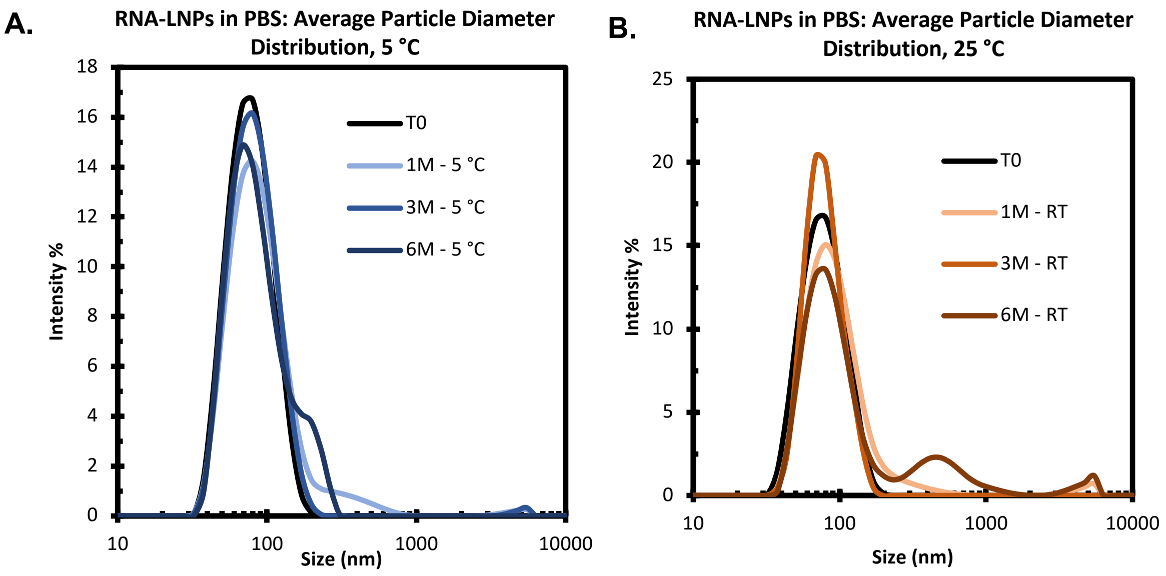

Supplementary Figure 2. Particle diameters of siRNA-LNPs in PBS buffer. Average (n=4) intensity data for size (diameter) distribution of PBS-based formulations at (A) 5 °C and (B) 25°C. Intensity-based scattering exhibited multimodal size distribution, with populations at 70-100 nm and >5 μm.

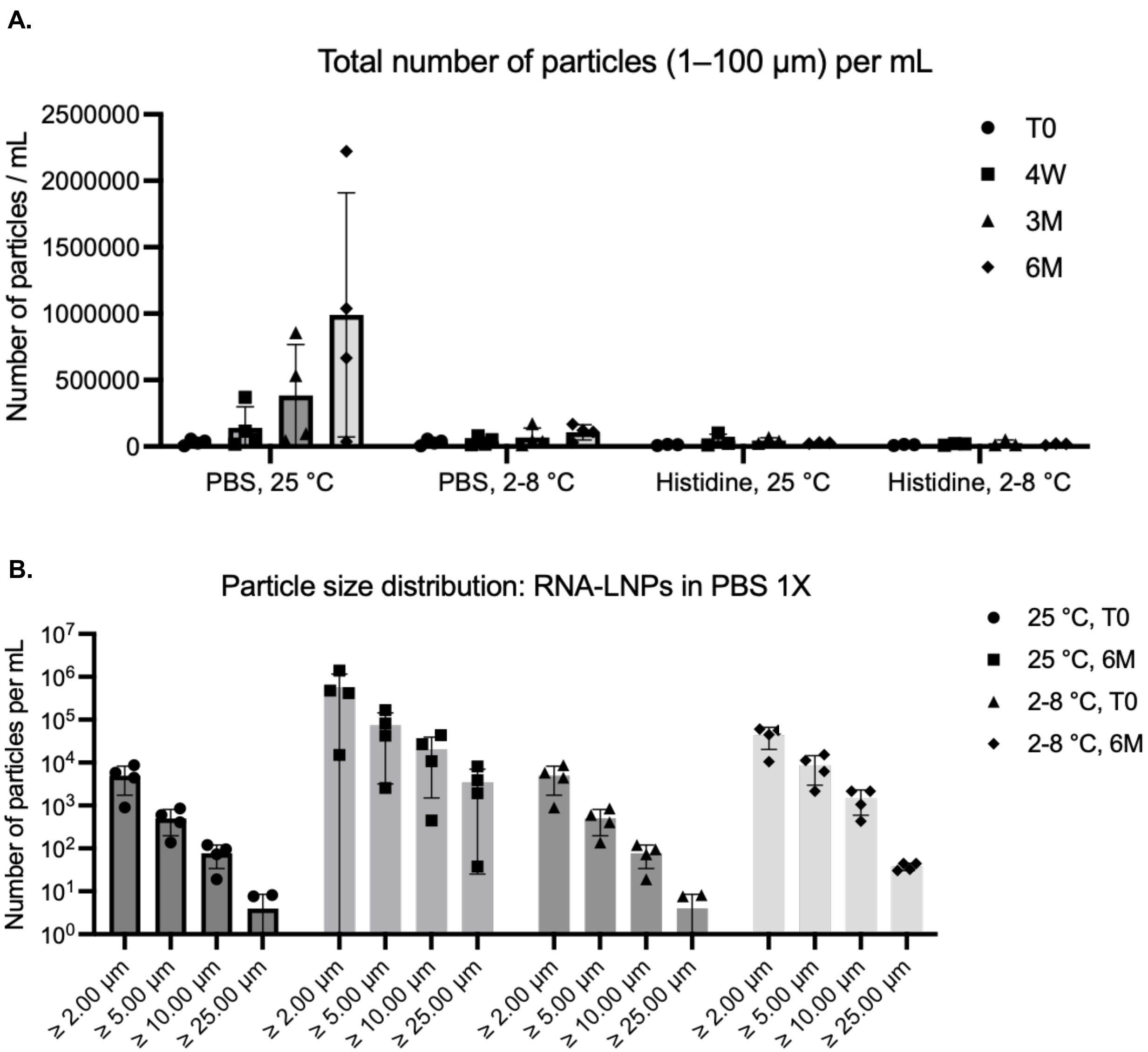

Supplementary Figure 3. Subvisible particle diameter analysis of siRNA-LNPs. MFI counts of the (A) total number of particles in PBS 1X and 10 mM histidine 140 mM NaCl ("His") formulations as well as the (B) particle size (diameter) distribution of the PBS formulations. Bar height and error bars represent the mean and standard deviation of n=4 biological replicates for PBS samples, n=3 biological replicates for histidine samples. Individual data points are plotted as closed symbols.

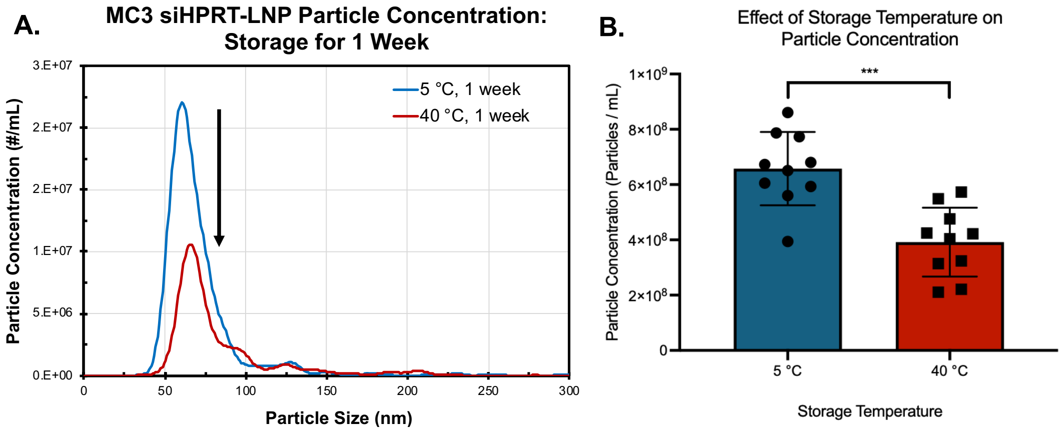

Supplementary Figure 4. Nanoparticle tracking analysis of siHPRT-LNPs in PBS 1X for 1 week at either 5 or 40 °C. Aliquots for each replicate and temperature are derived from the same master stock solution. (A) Particle concentration by size for LNPs held at 5 °C (blue line) or 40 °C (red line). No meaningful count of particles between 300–1000 nm was detected (data omitted). (B) Particle concentration (<1000 nm) for siHPRT-LNPs held at 5 and 40 °C. Bar height and error bars represent the mean and standard deviation of n=10 technical replicates. Individual data points are plotted as closed symbols. Significance is determined by a two-tailed Student's t-test of unequal variance, $p \leq 0.001$ ***.

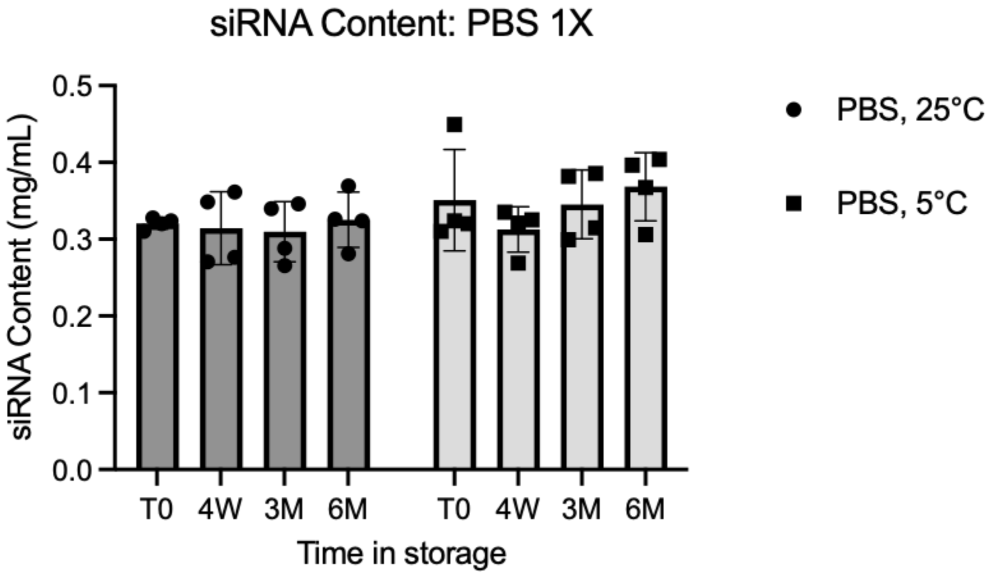

Supplementary Figure 5. RNA content of siHPRT-LNPs in PBS 1X at 4°C and room temperature for initial time (T0), 4 weeks, 3 months, and 6 months. RNA content determined by RiboGreen assay. Bar height and error bars represent the mean and standard deviation of n=4 biological replicates, with individual data points plotted as closed symbols. RNA content over time at 5 °C (closed circles) and 25 °C (closed squares) was evaluated by linear regression analysis and had ANOVA p-values of 0.38 and 0.85, respectively.

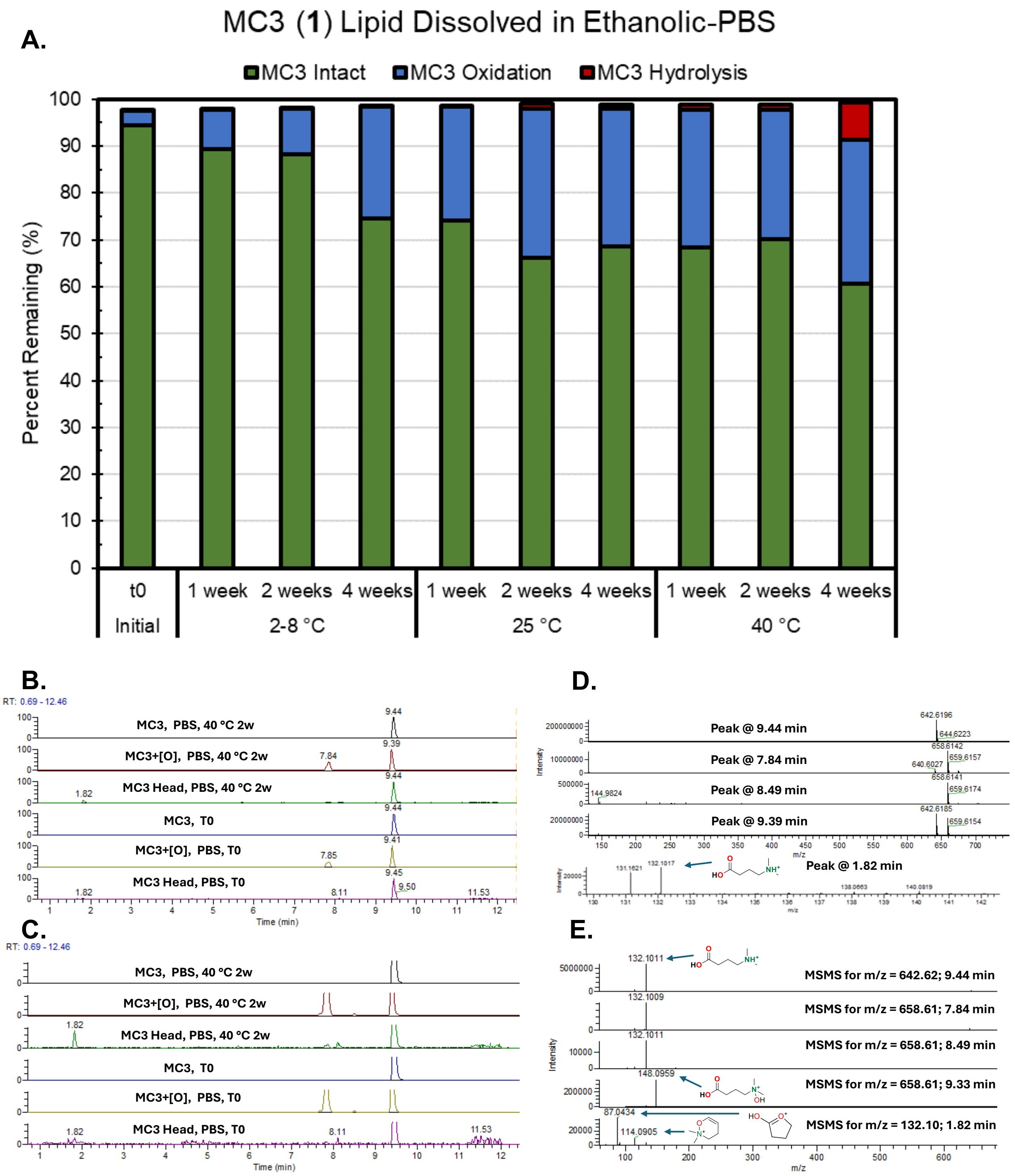

Supplementary Figure 6. Monitoring degradation of MC3 lipid in ethanolic solution. (A) Lipid integrity of MC3 **1** within ethanolic-buffer solutions at 5, 25 or 40 °C for 0, 1, 2 and 4 weeks. Percentage of MC3 left intact (green), with byproducts further broken down into percentage of MC3 oxidized (blue) and percentage of MC3 hydrolysis (red). Each time point represents an independent sample and stability pull. (B) Extracted ion chromatograms of MC3, mono-oxidized MC3, and MC3 heat ions for the control sample (T0) and the stressed sample at

40 °C for 2 weeks; (C) The expanded XICs; (D) Mass spectra of each XIC peak for the stressed sample at 40 ° C for 2 weeks; and (E) Tandem mass spectra of MC3 intact ion at m/z = 642.62, mono-oxidized MC3 ions at m/z = 658.61 and MC3 head ion at m/z = 132.10. MSMS results showed that mono-oxidized MC3 ions at 7.84 and 8.49 min contained the intact MC3 head ion, i.e. MC3 tails were oxidized, while mono-oxidized MC3 ion at 9.33 min contained oxidized MC3 head.

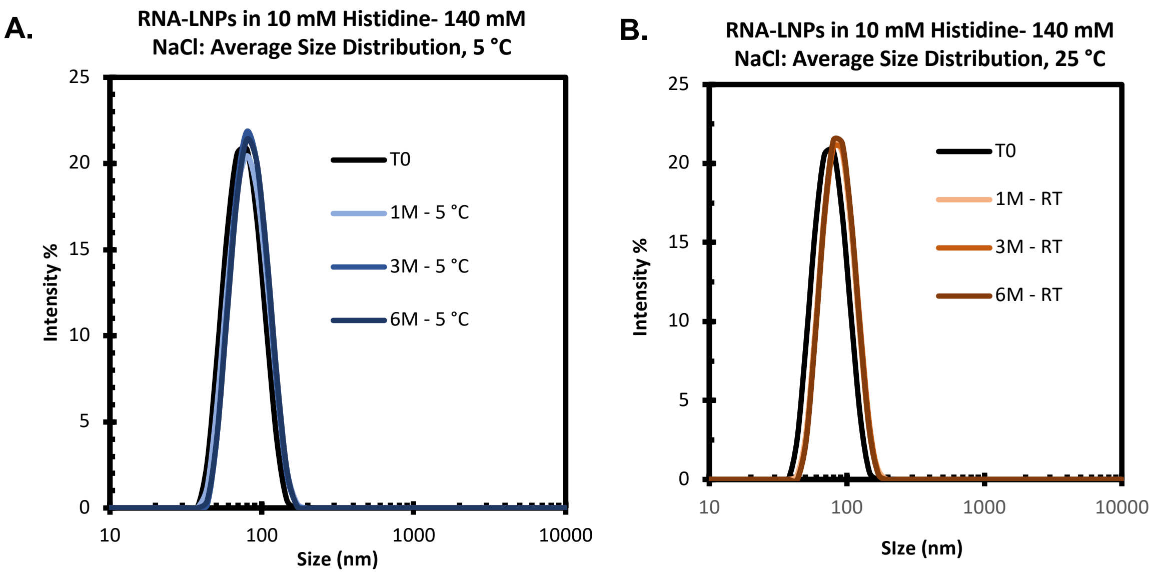

Supplementary Figure 7. Sizes of siRNA-LNPs in Histidine buffer. Average (n=3) intensity data for size distribution of histidine-based formulations at (A) 5°C and (B) 25°C. Intensity-based scattering exhibited a monomodal size distribution with populations at 70-100 nm.

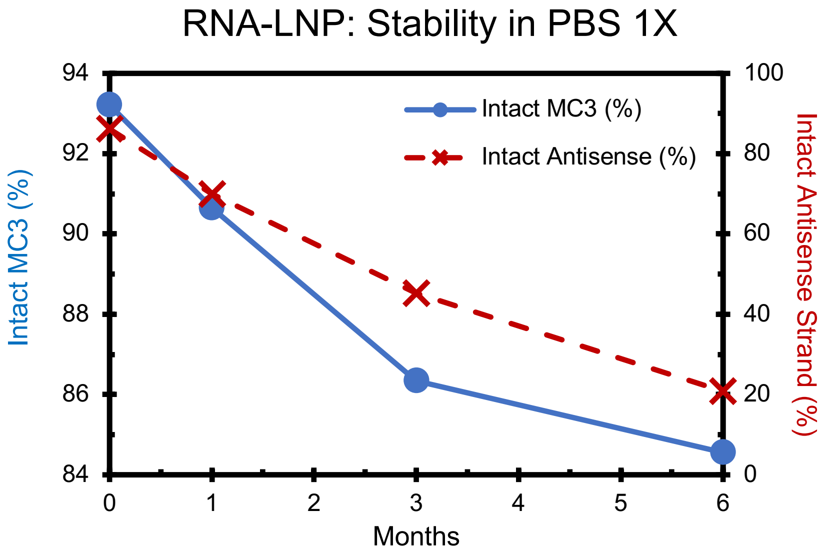

Supplementary Figure 8. Correlation between amount of intact MC3 (1) lipid and intact antisense strand of siHPRT within siRNA-LNPs. Data shown was generated from an RNA-LNP formulation stored in PBS 1X over 6 months. Triplicate data is shown in Figures 2F and 2G.

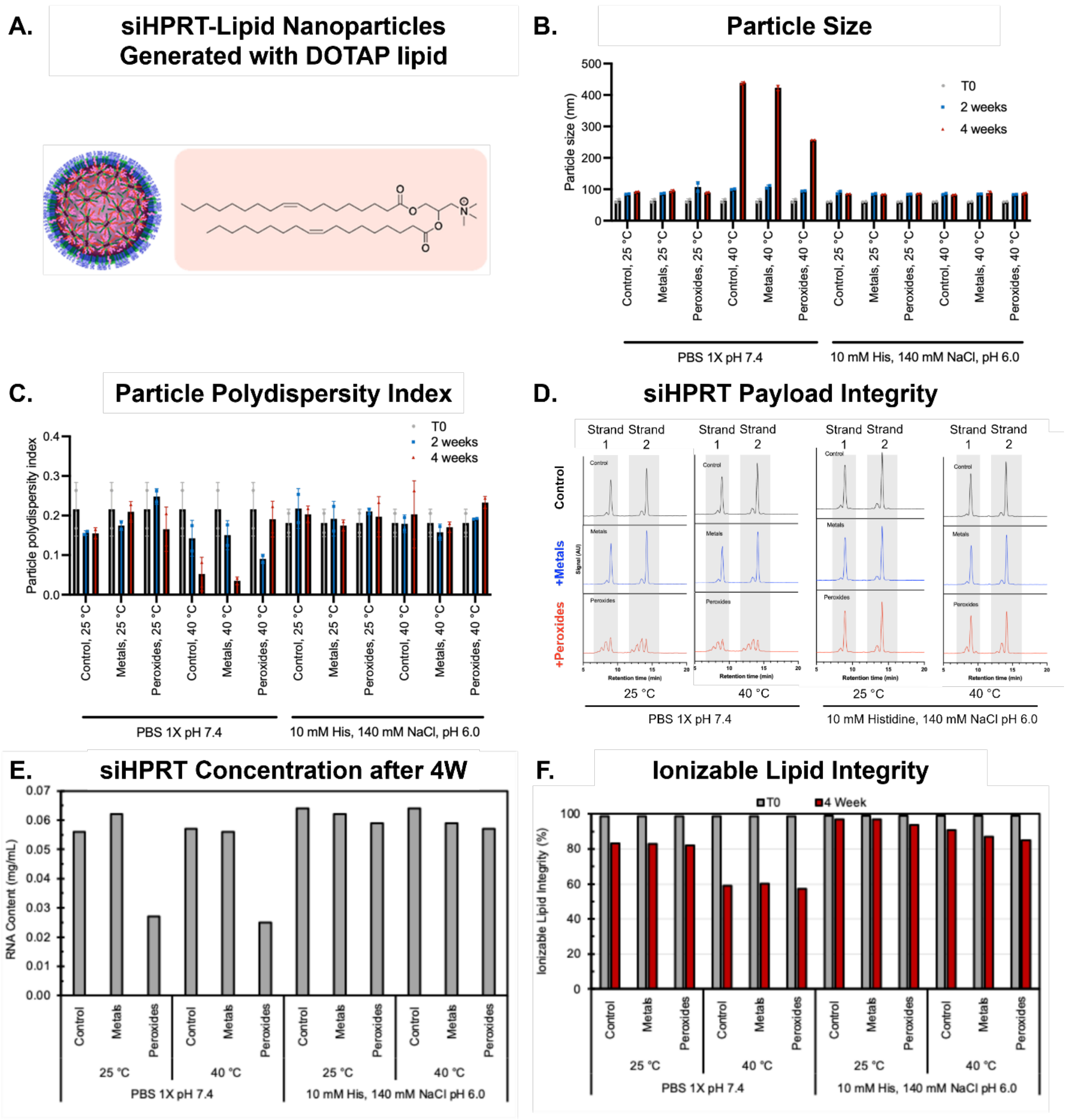

Supplementary Figure 9. (A) Analysis of DOTAP-stabilized siRNA-LNPs after four weeks across two temperatures and stressed conditions in two storage buffers: (i) PBS 1X pH 7.4 and (ii) 10 mM histidine + 140 mM NaCl, pH 6.0. Colloidal stability was analyzed via (B) particle size analysis and (C) polydispersity index by DLS. Bar height and error bars represent the mean and standard deviation of n=2 technical replicates, with each replicate individually plotted as a closed symbol. DOTAP LNPs demonstrate particle size increases in PBS at 40 °C but retain sizes below <100 nm in histidine buffer. Payload stability was analyzed via RP-IP-UPLC and is demonstrated via (D) chromatograms of both strands and (E) corresponding RNA content. Payload integrity is degraded by ~60% when particles are stored in PBS with peroxides regardless of temperature, while particles in histidine buffer retain payload integrity >90% under the same conditions. (F) Ionizable lipid integrity analyzed via LC-MS. The amount of intact

DOTAP lipid is degraded by ~40% when stored in PBS at 40 °C but integrity is retained >85% in histidine buffer under the same conditions. Note that nearly all of the DOTAP degradation is attributed to hydrolysis (*e.g.,* PBS control at 40 °C is 41.1% degraded, with 40.8% corresponding to hydrolytic cleavage products and 0.3% corresponding to oxidative degradants). The identity and relative amounts of each DOTAP degradant is provided within Supplementary Table 3. See Materials and Methods for detailed information on RNA-LNP formation procedures, DLS, LC-MS, and HPLC analysis.

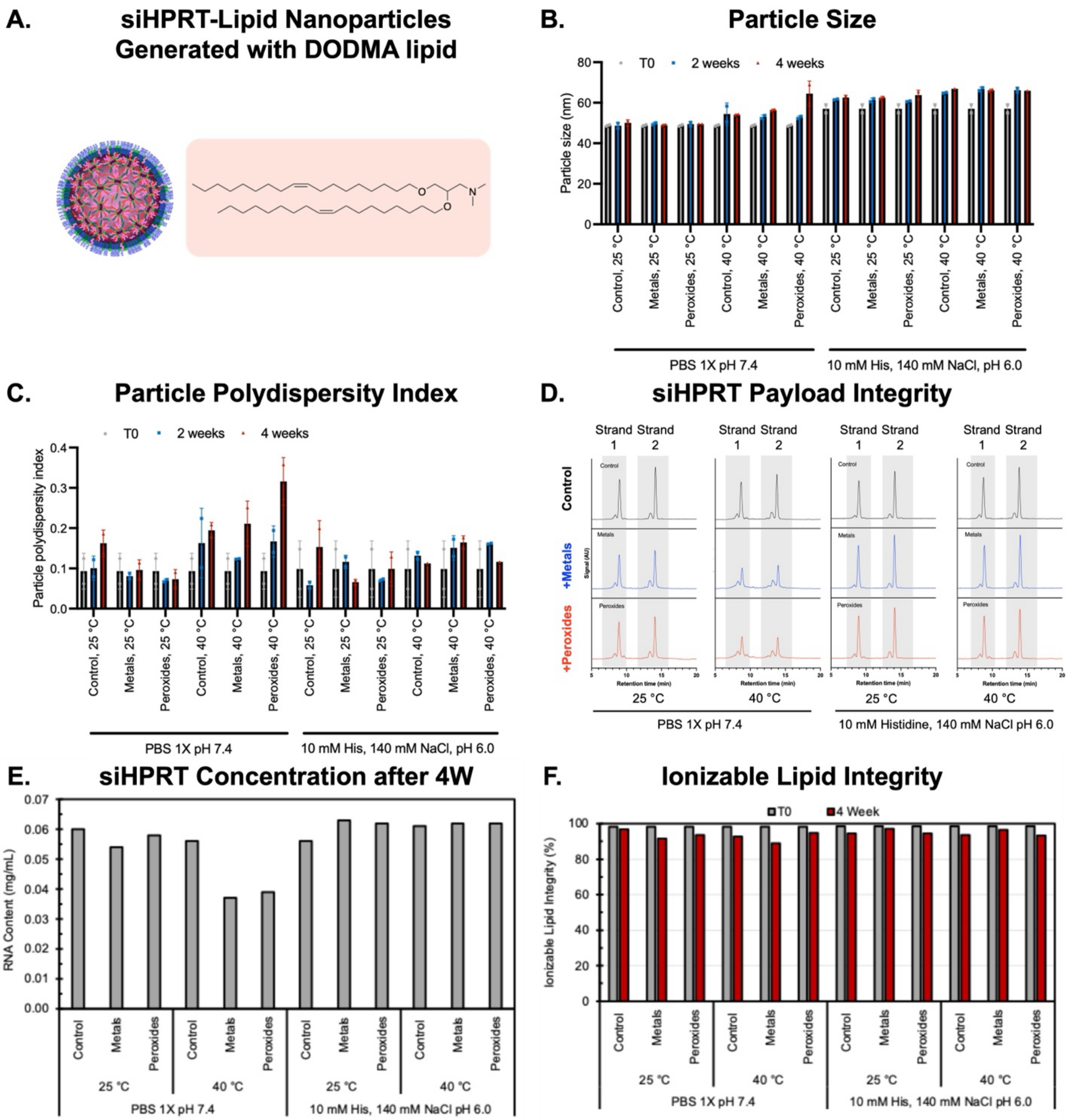

Supplementary Figure 10. (A) Analysis of DODMA-stabilized siRNA-LNPs after four weeks across two temperatures and stressed conditions in two storage buffers: (i) PBS 1X pH 7.4 and (ii) 10 mM histidine + 140 mM NaCl, pH 6.0. Colloidal stability was analyzed via (B) particle size analysis and (C) polydispersity index (PDI) by DLS. Bar height and error bars represent the mean and standard deviation of n=2 technical replicates, with each replicate individually plotted as a closed symbol. DODMA LNPs retain particles sizes below <100 nm in both PBS and histidine buffer but PDI is increased slightly to >0.3 when stored in PBS + peroxides. Payload stability was analyzed via RP-IP-UPLC and is demonstrated via (D) chromatograms of both strands and (E) corresponding RNA content. Payload integrity is degraded by ~40% when particles are stored in PBS with metals or peroxides at 40 °C, while

particles in histidine buffer retain payload integrity >90% under the same conditions. (F) Ionizable lipid integrity analyzed via LC-MS. The amount of intact DODMA lipid retained >85% in all buffers and conditions. The identity and relative amounts of each DODMA degradant is provided within Supplementary Table 4. See Materials and Methods for detailed information on RNA-LNP formation procedures, DLS, LC-MS, and HPLC analysis.

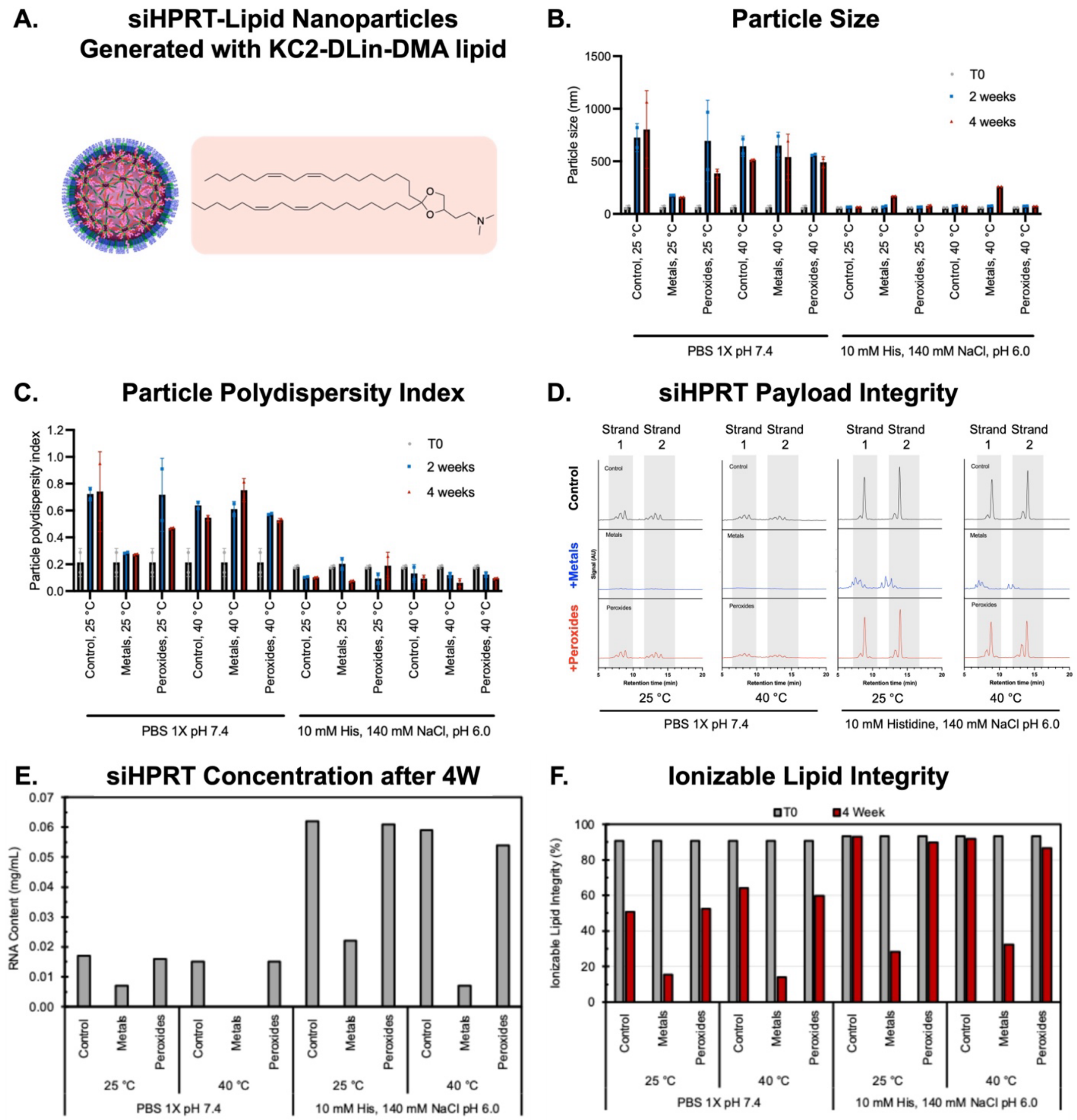

Supplementary Figure 11. (A) Analysis of DLin-KC2-DMA-stabilized siRNA-LNPs after four weeks across two temperatures and stressed conditions in two storage buffers: (i) PBS 1X pH 7.4 and (ii) 10 mM histidine + 140 mM NaCl, pH 6.0. Colloidal stability was analyzed via (B) particle size analysis and (C) polydispersity index by DLS. Bar height and error bars represent the mean and standard deviation of n=2 technical replicates, with each replicate individually plotted as a closed symbol. DLin-KC2-DMA LNPs demonstrate large particle size and polydispersity increases in PBS at either 25 or 40 °C. Storage in histidine buffer mitigates these size changes and retains particle sizes <75 nm in all conditions except for +metals at 40 °C for 4 weeks. Payload stability was analyzed via RP-IP-UPLC and is demonstrated via (D) chromatograms of both strands and (E) corresponding RNA content. With or without peroxide

spiking, payload integrity falls below 30% when particles are stored in PBS regardless of temperature or stress, while particles in histidine buffer retain payload integrity >87% under the same conditions. However, histidine does not demonstrate sufficient payload protection against the severe degradation that metals induce. (F) Ionizable lipid integrity analyzed via LC-MS for the major DLin-KC2-DMA variant observed (-28 Da compared to the expected MW implies two methylenes shorter than advertised). With or without peroxide spiking, the amount of intact lipid falls to ~50–64% in PBS; conversely, ionizable lipid integrity is retained >85% in histidine buffer under the same conditions. However, histidine buffer cannot efficiently protect against metal spiking, with integrity <35% regardless of buffer. The identity and relative amounts of each DLin-KC2-DMA degradant is provided within Supplementary Table 5. See Materials and Methods for detailed information on RNA-LNP formation procedures, DLS, LC-MS, and HPLC analysis.

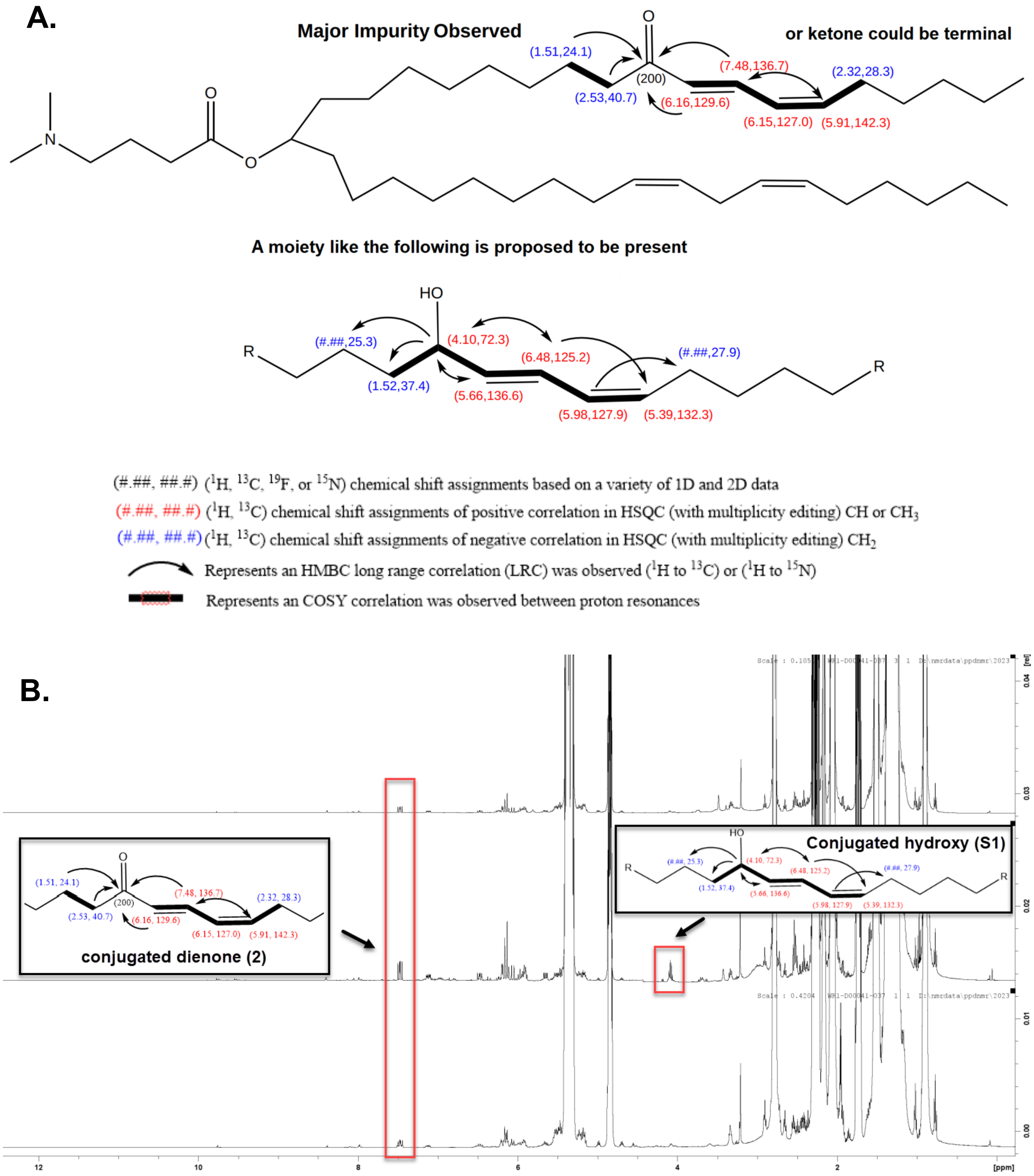

Supplementary Figure 12. Structure elucidation of MC3 degradants by 1H-NMR. (A) Major and minor impurities observed within MC3 raw materials, a conjugated *E,Z*-dienone (**2**) and dienol (**S1**) species, respectively. (B) ^{1}H-NMR overlay of DLin-MC3-DMA (**1**) from three manufacturers and the structure elucidation of oxidative degradants **2** and **S1**.

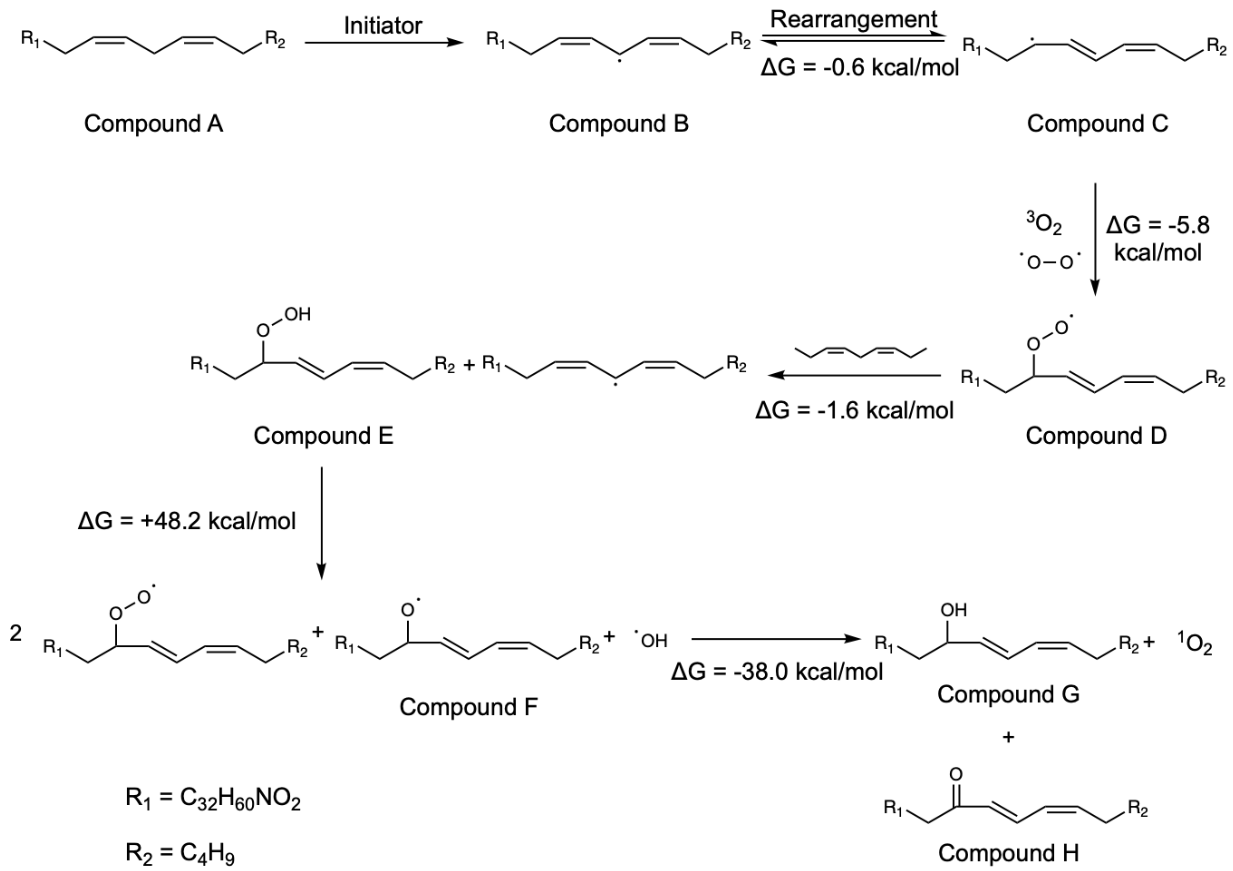

Supplementary Figure 13. Proposed mechanism of MC3 oxidation and observed byproducts, with free energy values determined by density functional theory. Compound letters refer to atomic coordinates that are included within Supplementary Data 1–8 as plain, unformatted text files.

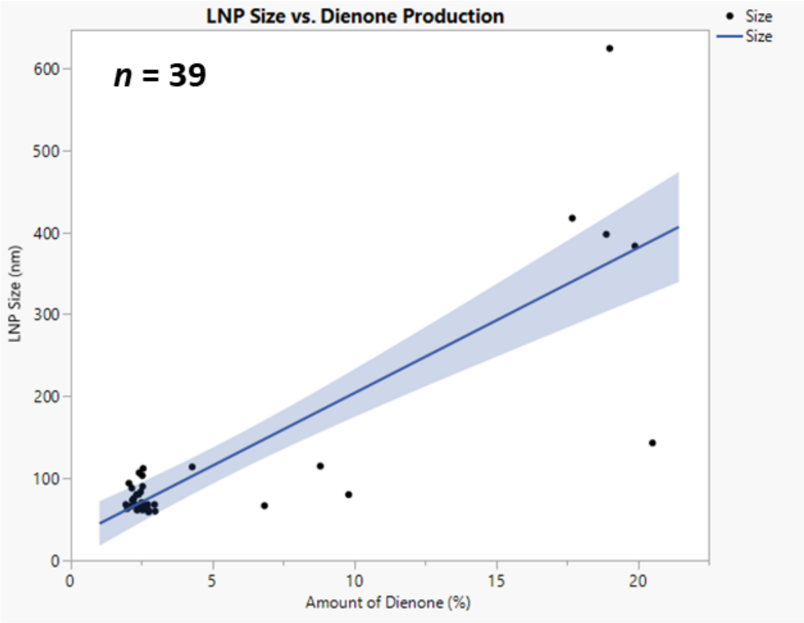

Supplementary Figure 14. Correlation between LNP particle size and the amount of dienone byproduct in the formulation.

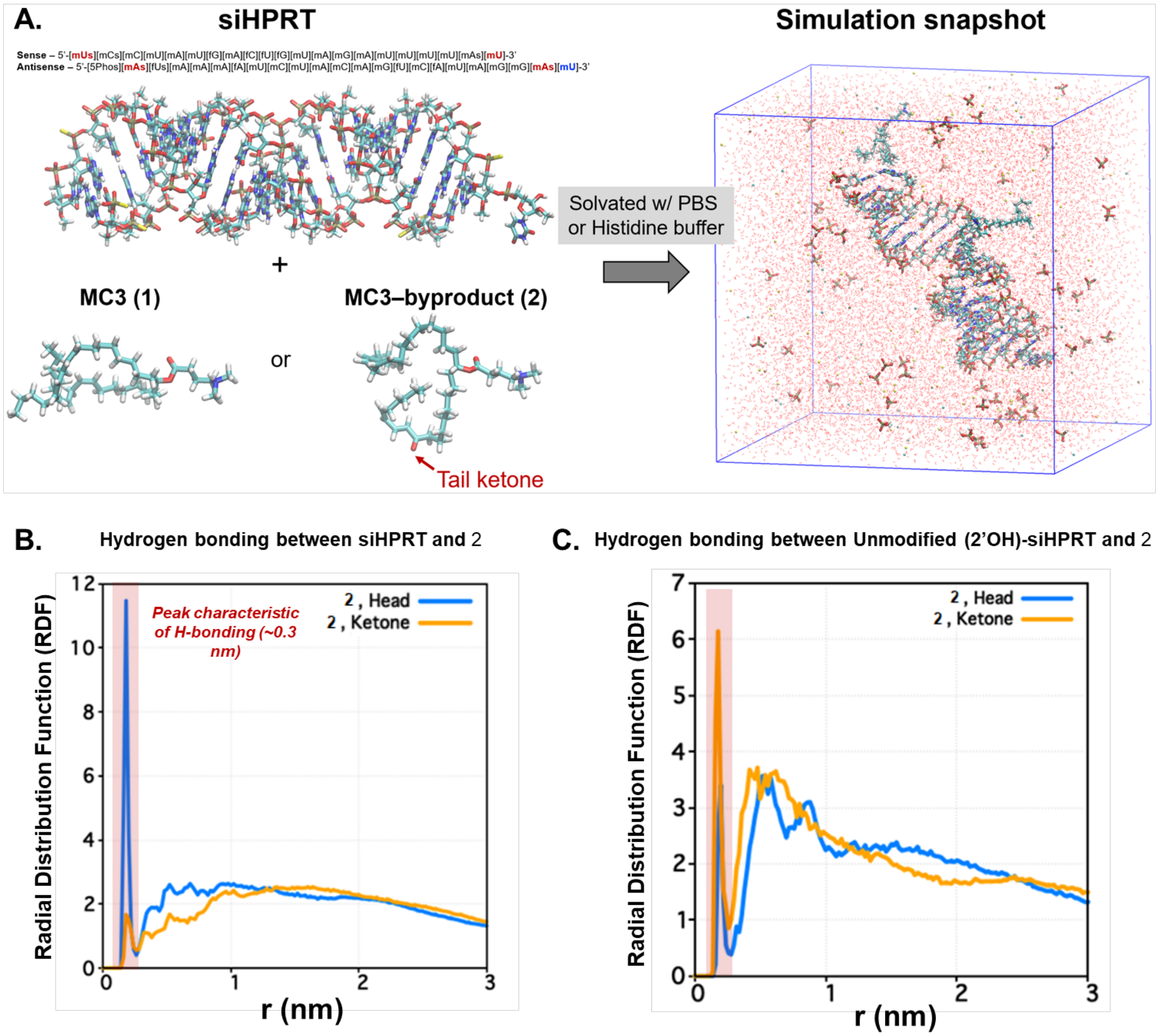

Supplementary Figure 15. Modeling non-covalent interactions between siHPRT and MC3's E,Z-dienone degradation product. (A) Sequence of the sense and antisense strands of siHPRT cargo (legend below), along with the ball and stick models of MC3 (**1**) or dienone byproduct (**2**). After molecular dynamics simulations, radial distribution functions (RDF) are determined for **2**'s interaction with (B) modified (2'OMe) or (C) unmodified (2'OH) RNA via either **2**'s head group amine (blue line) or tail ketone (orange line). Legend: Upper case letters indicate identity of the nucleoside (U: uracil; C: cytosine; A: adenine; G: guanine). Lower case letters in front of the nucleoside represent modifications to that nucleoside at the 2' position (mX: 2'-*O*-methyl ribonucleoside; fX: 2'-fluoro-deoxyribonucleoside). Lower case letters after the nucleoside represent modifications to the phosphate linkage (Xs: phosphorothioate linkage).

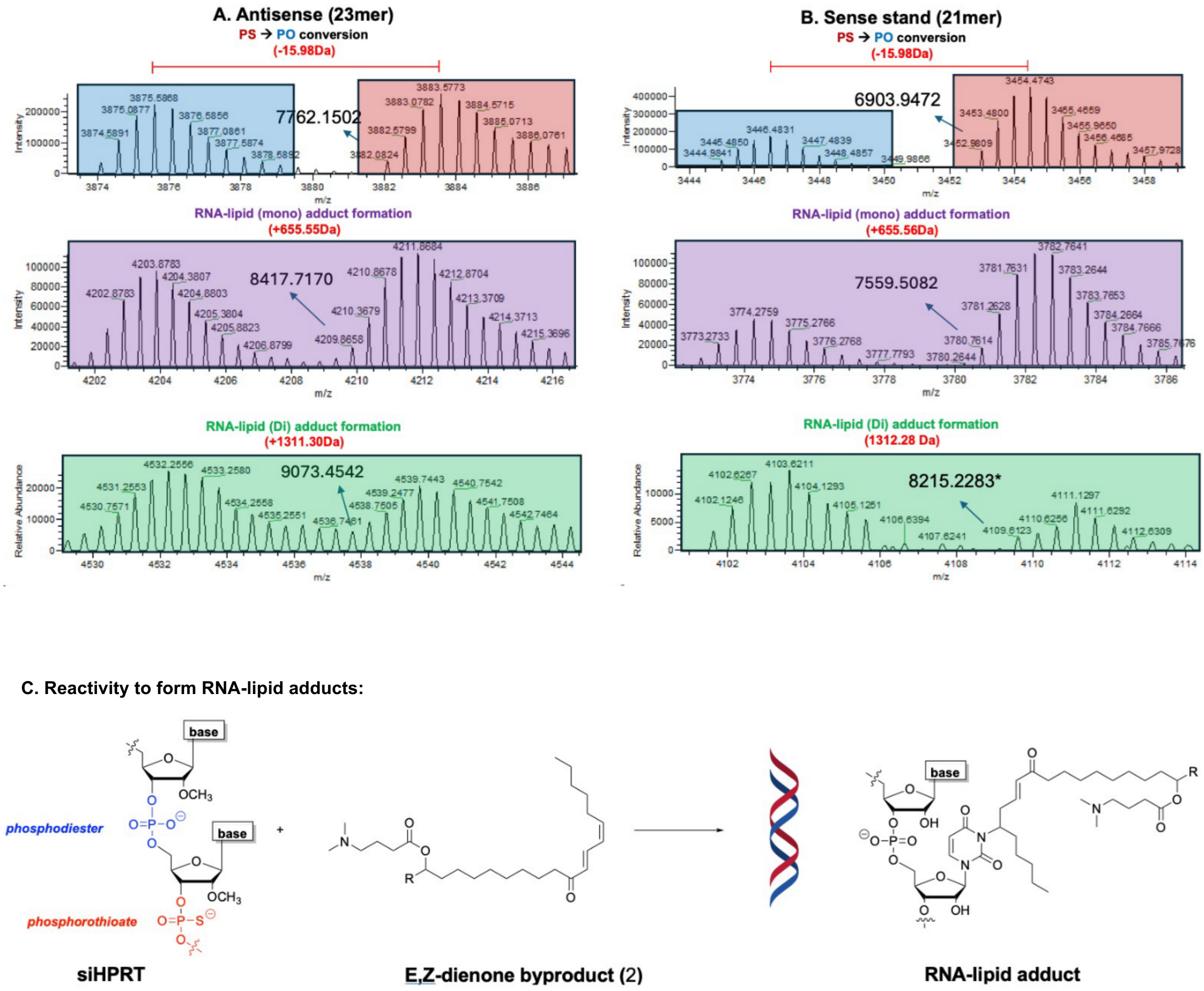

Supplementary Figure 16. LC/MS analysis of siHPRT cargo after extraction from stressed RNA-LNPs stored in PBS 1X. Molecular weight distributions of (A) antisense strand and (B) sense strand, both native (top), 1x lipidated strand (middle) and 2x lipidated strand (bottom). (C) Illustrations of proposed siRNA degradation mechanisms, namely PS → PO conversion and lipidation by reaction of nucleobases with dienone byproduct **2**.

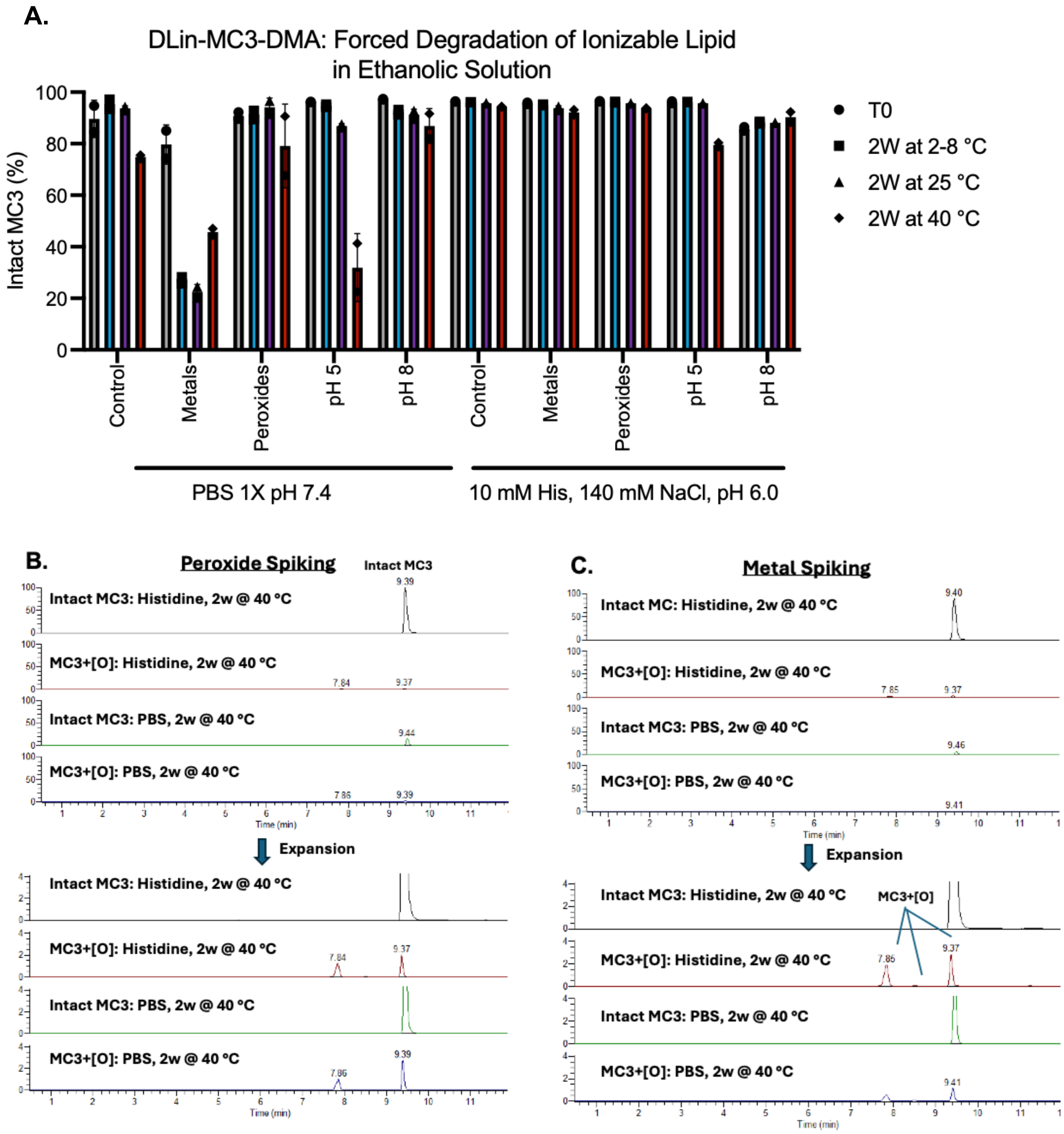
A.
DLin-MC3-DMA: Forced Degradation of Ionizable Lipid in Ethanolic Solution
Intact MC3 (%)
100
80
60
40
20
0
Control
Metals
Peroxides
pH 5
pH 8
PBS 1X pH 7.4
10 mM His, 140 mM NaCl, pH 6.0
T0
2W at 2-8 °C
2W at 25 °C
2W at 40 °C
B.
Peroxide Spiking
Intact MC3
Intact MC3: Histidine, 2w @ 40 °C
MC3+[O]: Histidine, 2w @ 40 °C
Intact MC3: PBS, 2w @ 40 °C
MC3+[O]: PBS, 2w @ 40 °C
9.39
7.84
9.37
9.44
7.86
Time (min)
Expansion
C.
Metal Spiking
Intact MC: Histidine, 2w @ 40 °C
MC3+[O]: Histidine, 2w @ 40 °C
Intact MC3: PBS, 2w @ 40 °C
MC3+[O]: PBS, 2w @ 40 °C
Intact MC3: Histidine, 2w @ 40 °C
9.40
7.85
9.37
9.46
9.41
7.86
MC3+[O]
Time (min)
Expansion

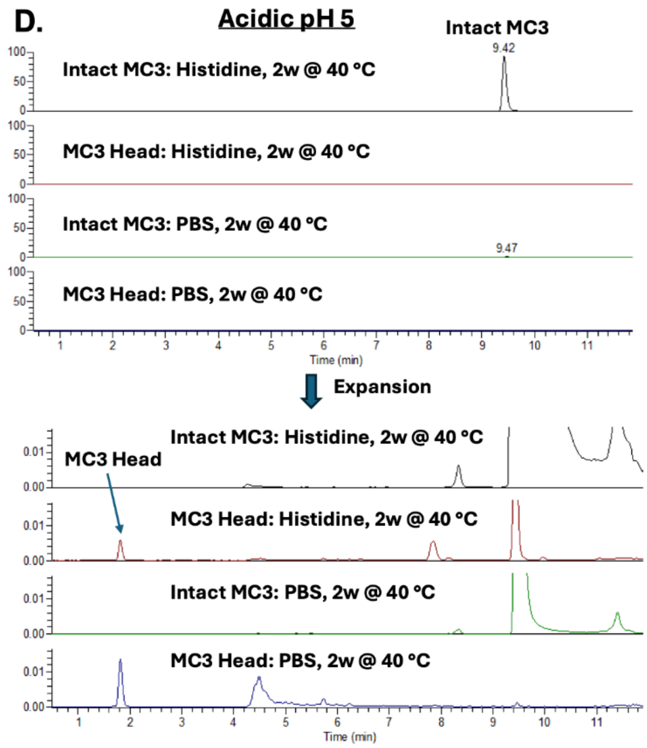

Supplementary Figure 17. (A) Degradation of MC3 1 in solution under forced degradation conditions, *i.e.*, (i) inclusion of metals (1 ppm Ni, Fe and Cu), (ii) peroxides (1 ppm H_2O_2), (iii) acidic and (iv) basic formulations. The two solutions compared were 25 volume percent ethanol to solubilize MC3 and either PBS 1X or histidine (10 mM histidine + 140 mM NaCl, pH 6.0). Bar height and error bars represent the mean and standard deviation of n=2 technical replicates. Individual data points are plotted as closed symbols. (B–D) Extracted ion chromatograms for stressed sample at 40 °C for 2 weeks, including expanded XICs of MC3 and (B,C) oxidized (MC3+[O]") via (B) peroxide or (C) metal spiking, or (D) hydrolyzed ("MC3 Head") MC3 ions. Plots are on the same scale, and intact MC3 intensity in PBS is <10% of that in Histidine.

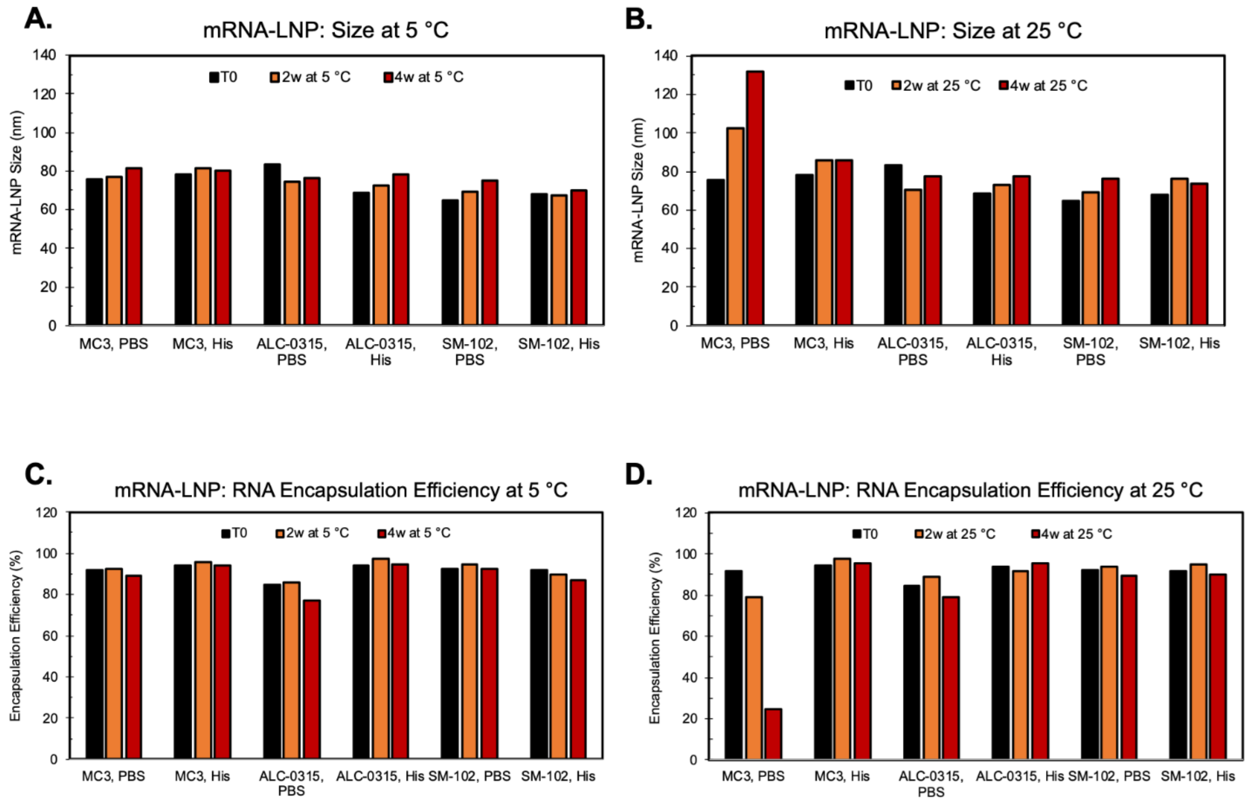

Supplementary Figure 18. Stability of EPO mRNA-LNPs formulated in phosphate- ("PBS") or histidine-containing ("His") buffer using three different ionizable lipids: MC3 **1**, ALC-0315 or SM-102. Particle size of mRNA-LNPs was evaluated at (A) 5 °C or (B) 25 °C. Encapsulation efficiency of mRNA-LNPs was evaluated at (C) 5 °C or (D) 25 °C. Each time point represents an independent sample and stability pull. Legend: initial time (black bars), 2 weeks at storage temperature (orange bars), 4 weeks at storage temperature (red bars). Lipid integrity of each formulation is included within Supplementary Table 8.

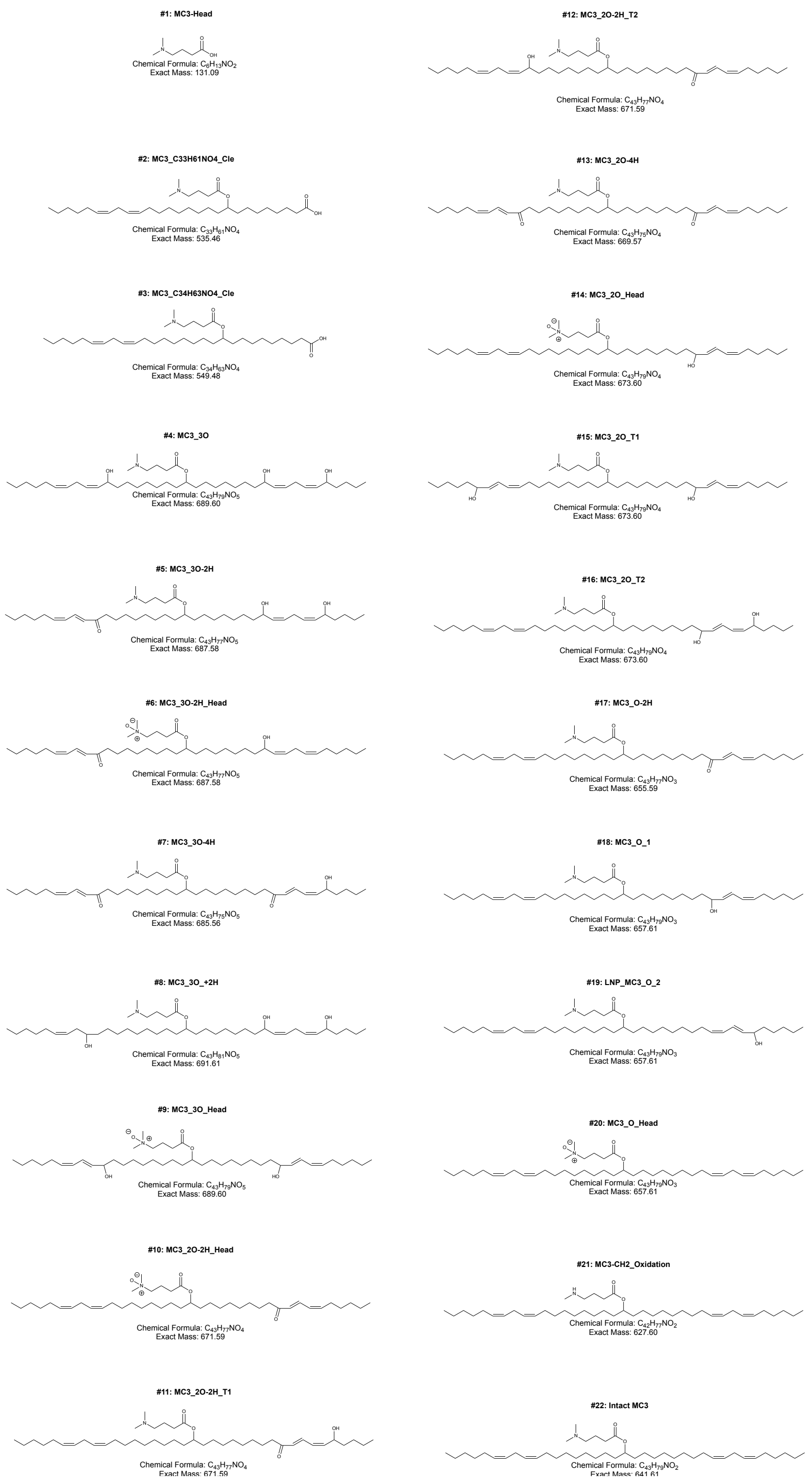

Supplementary Figure 19. Structures which match the masses identified through LC/MS analysis of DLin-MC3-DMA (**1**)-containing solutions described herein. Note that structures shown are hypothetical, except for intact MC3 (1, #22), identified dienone (2, #17) and dienol (S1, #18).

Supplementary Tables

Supplementary Table 1. LC/MS analysis of MC3 lipid and degradants after siHPRT-LNP storage over six months at 5 or 25 °C in two different buffers: (i) histidine (10 mM), pH 6.0 or (ii) phosphate-buffered saline (PBS) 1X, pH 7.4. Selected data from this table is presented in Figure 2F, Figure 3F. Exemplary structures of each degradant can be found in Supplementary Figure 19.

siHPRT-LNPs		10 mM Histidine + 140 mM NaCl									PBS 1X								
Degradant (examples of structures found in Supplementary Figure 19)	Mass (Da)	t0	5 °C 1M	5 °C 2M	5 °C 3M	5 °C 6M	25 °C 1M	25 °C 2M	25 °C 3M	25 °C 6M	t0	5 °C 1M	5 °C 2M	5 °C 3M	5 °C 6M	25 °C 1M	25 °C 2M	25 °C 3M	25 °C 6M
Head group (cleaved at ester)	131.09	0.00	0.02	0.03	0.04	0.07	0.07	0.10	0.15	0.11	0.01	0.05	0.08	0.15	0.14	0.11	0.02	0.19	0.17
C33H61NO4_Cle	535.46	0.02	0.02	0.02	0.02	0.02	0.02	0.02	0.02	0.03	0.02	0.02	0.02	0.02	0.03	0.02	0.02	0.04	0.07
C34H63NO4_Cle	549.48	0.14	0.12	0.14	0.15	0.16	0.16	0.16	0.18	0.19	0.16	0.17	0.18	0.19	0.19	0.17	0.17	0.30	0.44
+3O, Triple Oxidation, all at Tail	689.60	0.02	0.02	0.03	0.03	0.03	0.04	0.03	0.04	0.05	0.05	0.05	0.05	0.09	0.06	0.07	0.05	0.12	0.15
+3O, Triple Oxidation, one at head, two at tail	689.60	0.01	0.01	0.01	0.02	0.02	0.02	0.02	0.03	0.05	0.03	0.02	0.02	0.04	0.03	0.04	0.03	0.06	0.09
+3O-2H, Triple Oxidation	687.58	0.00	0.00	0.00	0.00	0.00	0.00	0.00	0.00	0.01	0.00	0.00	0.00	0.00	0.01	0.01	0.01	0.02	0.03
+3O-2H: Triple Oxidation, one at head, two at tail	687.58	0.00	0.00	0.00	0.00	0.01	0.01	0.01	0.01	0.02	0.01	0.01	0.01	0.01	0.01	0.01	0.01	0.03	0.05
+3O-4H, Triple Oxidation	685.56	0.02	0.02	0.02	0.02	0.03	0.03	0.03	0.04	0.05	0.03	0.03	0.04	0.05	0.05	0.05	0.05	0.10	0.17
+3O_2H, Triple Oxidation	691.61	0.00	0.00	0.00	0.00	0.00	0.00	0.00	0.00	0.00	0.02	0.01	0.01	0.01	0.00	0.01	0.00	0.01	0.00
+2O-2H, Double oxidation all at Tail Position 1	671.58	0.01	0.01	0.01	0.02	0.02	0.02	0.02	0.03	0.04	0.02	0.02	0.02	0.03	0.03	0.03	0.02	0.06	0.09
+2O-2H, Double oxidation all at Tail Position 2	671.58	0.04	0.05	0.06	0.07	0.11	0.08	0.12	0.18	0.29	0.06	0.06	0.06	0.08	0.09	0.08	0.08	0.15	0.27
+2O-2H, Double Oxidation, one at head, one at tail	671.58	0.16	0.12	0.10	0.10	0.08	0.10	0.09	0.09	0.10	0.24	0.23	0.22	0.27	0.26	0.33	0.30	0.59	0.86
+2O-4H, Double Oxidation, all at Tail Position 1	669.57	0.04	0.05	0.05	0.06	0.10	0.07	0.10	0.14	0.23	0.05	0.06	0.07	0.09	0.10	0.09	0.08	0.18	0.30
+2O, Double Oxidation, all at Tail Position 1	673.60	0.06	0.07	0.09	0.12	0.12	0.14	0.13	0.16	0.16	0.22	0.19	0.18	0.34	0.19	0.25	0.10	0.33	0.25
+2O, Double Oxidation, all at Tail Position 2	673.60	0.01	0.01	0.02	0.02	0.04	0.02	0.04	0.05	0.10	0.02	0.02	0.02	0.03	0.02	0.02	0.02	0.05	0.08
+2O, Double Oxidation, one at head, one at tail	673.60	0.10	0.11	0.11	0.12	0.12	0.12	0.12	0.12	0.13	0.14	0.18	0.20	0.29	0.29	0.27	0.26	0.50	0.75
+O, Mono Oxidation, at Tail Position 1	657.61	1.63	2.42	2.55	2.87	2.55	2.85	2.69	2.98	2.10	3.67	4.34	4.30	5.17	4.75	4.89	2.32	6.40	6.27
+O, Mono Oxidation, at Tail Position 2	657.61	0.53	0.84	0.94	1.11	1.17	1.13	1.28	1.62	1.54	1.10	1.31	1.34	1.68	1.61	1.51	0.91	2.29	2.69
+O, Mono Oxidation, at Head	657.61	0.16	0.16	0.16	0.17	0.17	0.18	0.18	0.19	0.20	0.20	0.21	0.21	0.25	0.22	0.23	0.19	0.32	0.39
+O-2H, Mono Oxidation, at Tail	655.59	0.55	0.59	0.64	0.70	0.99	0.75	1.00	1.26	2.36	0.51	0.57	0.60	0.69	0.77	0.68	0.86	1.10	1.69
CH2_Oxidation, Oxidation and lost Methyl at Head	627.59	0.07	0.13	0.15	0.17	0.18	0.21	0.22	0.30	0.27	0.24	0.30	0.33	0.42	0.52	0.48	0.23	0.83	0.63
Intact MC3	641.61	96.42	95.22	94.85	94.19	94.00	93.99	93.65	92.40	91.96	93.21	92.12	92.04	90.09	90.63	90.64	94.26	86.34	84.56

Supplementary Table 2. LC/MS analysis of siHPRT, degradants, and siHPRT-lipid adduct formation after siHPRT-LNP storage over six months at 5 or 25 °C in two different buffers: (i) histidine (10 mM), pH 6.0 or (ii) phosphate-buffered saline (PBS) 1X, pH 7.4. Selected data from this table is presented in Figure 2G,H and Figure 3G,H. Legend: SS: sense strand; AS: antisense strand. First values represent the number of nucleosides present in the byproduct and where cleavage occurred (*e.g.,* "SS: 1-19" indicates that the last two nucleotides were cleaved, while "SS: 2-21" indicates that the first nucleotide was cleaved). Unless otherwise described, degradants result in a 5'-OH. In other instances, the phosphate is retained at the 5' position and is denoted with a "P" (*e.g.,* "P-2-21"). Number of phosphorothioate-to-phosphodiester transformations are denoted by "#x PS-PO." Defluorination of 2'-fluoro-deoxyribonucleosides is denoted as "#x loss F." The number of RNA-lipid adducts (reactivity between **1** and **2**, as described in Figure 4) is denoted by "#x Lipid Adducts."

	siHPRT-LNPs in 10 mM Histidine + 140 mM NaCl									siHPRT-LNPs in PBS 1X								
siHPRT RNA Degradant	T0	5 °C, 1M	5 °C, 2M	5 °C, 3M	5 °C, 6M	25 °C, 1M	25 °C, 2M	25 °C, 3M	25 °C, 6M	T0	5 °C, 1M	5 °C, 2M	5 °C, 3M	5 °C, 6M	25 °C, 1M	25 °C, 2M	25 °C, 3M	25 °C, 6M
SS: 1-19, 1x PS-PO	0.00	0.00	0.00	0.00	0.00	0.00	0.00	0.00	0.01	0.00	0.00	0.00	0.00	0.00	0.00	0.00	0.00	0.08
SS: 1-19	0.01	0.01	0.01	0.01	0.00	0.00	0.02	0.00	0.00	0.00	0.00	0.01	0.00	0.00	0.00	0.01	0.00	0.65
SS: 2-21, 2x PS-PO	0.00	0.00	0.00	0.00	0.00	0.00	0.00	0.00	0.00	0.00	0.00	0.00	0.00	0.00	0.00	0.00	0.00	0.21
SS: 2-21, 1x PS-PO	0.00	0.01	0.01	0.01	0.02	0.01	0.03	0.03	0.10	0.01	0.02	0.02	0.04	0.08	0.08	0.04	0.03	1.57
SS: 2-21	0.39	0.50	0.34	0.38	0.65	0.40	0.67	0.72	0.97	0.77	0.78	0.74	1.01	1.37	1.32	1.74	1.87	3.23
SS: P-2-21, 1x PS-PO	0.01	0.04	0.02	0.03	0.06	0.01	0.00	0.00	0.00	0.05	0.01	0.01	0.00	0.01	0.00	0.02	0.00	0.29
SS: P-2-21	0.03	0.04	0.02	0.03	0.06	0.03	0.08	0.09	0.14	0.07	0.06	0.06	0.06	0.13	0.12	0.15	0.06	0.40
SS: P-3-21, 1x PS-PO	0.00	0.00	0.00	0.00	0.00	0.00	0.00	0.00	0.00	0.00	0.00	0.00	0.00	0.00	0.00	0.00	0.00	0.05
SS: P-3-21	0.00	0.00	0.00	0.00	0.00	0.00	0.00	0.01	0.02	0.00	0.00	0.00	0.00	0.03	0.00	0.00	0.00	0.12
SS: 3-21	0.01	0.01	0.00	0.01	0.01	0.00	0.05	0.00	0.04	0.00	0.00	0.00	0.00	0.00	0.00	0.00	0.00	0.29
SS: 3x PS-PO	0.00	0.00	0.00	0.00	0.00	0.00	0.00	0.00	0.00	0.00	0.00	0.00	0.00	0.03	0.04	0.01	0.02	1.06
SS: 2x PS-PO	0.09	0.22	0.08	0.19	0.39	0.04	0.51	0.54	1.87	0.28	0.26	0.22	0.30	0.61	0.61	0.87	1.07	11.05
SS: 1x PS-PO	6.52	7.40	7.63	8.27	11.49	9.75	13.21	14.91	22.01	8.44	6.05	6.63	5.69	13.51	13.84	21.45	28.05	38.07
SS Intact	92.94	91.76	91.88	91.07	87.32	89.75	85.43	83.70	74.84	90.38	92.82	92.32	92.89	84.22	83.98	75.70	68.89	42.93
AS: 1-21, 2x PS-PO	0.00	0.00	0.00	0.00	0.00	0.01	0.01	0.02	0.03	0.00	0.00	0.01	0.01	0.04	0.06	0.16	0.36	1.53
AS: 1-21, 2x PS-PO, 1x loss F	0.02	0.02	0.03	0.03	0.07	0.05	0.09	0.17	0.26	0.02	0.10	0.17	0.21	0.39	0.58	1.04	1.54	2.27
AS: 1-21, 1x PS-PO	0.02	0.04	0.05	0.05	0.11	0.09	0.13	0.24	0.36	0.04	0.15	0.24	0.28	0.58	0.84	1.45	2.22	3.42
AS: 1-21, 1x PS-PO, 1x loss F	0.05	0.09	0.10	0.11	0.19	0.18	0.21	0.30	0.27	0.06	0.16	0.19	0.22	0.27	0.30	0.37	0.41	0.50
AS: 2-23, 3x PS-PO	0.00	0.00	0.00	0.00	0.00	0.00	0.00	0.01	0.02	0.00	0.00	0.00	0.00	0.01	0.03	0.03	0.10	0.75
AS: 2-23, 2x PS-PO	0.02	0.03	0.03	0.03	0.06	0.05	0.07	0.15	0.32	0.03	0.04	0.06	0.06	0.13	0.16	0.34	0.61	1.54
AS: 2-23, 1x PS-PO	0.67	0.62	0.56	0.53	0.62	0.64	0.56	0.61	0.72	0.93	0.81	0.79	0.69	0.71	0.74	0.68	0.72	0.66
AS: 4x PS-PO	0.00	0.00	0.00	0.00	0.00	0.00	0.00	0.00	0.00	0.00	0.00	0.00	0.00	0.00	0.00	0.01	0.05	0.54
AS: 3x PS-PO	0.00	0.01	0.01	0.01	0.03	0.03	0.05	0.12	0.53	0.01	0.03	0.03	0.03	0.09	0.18	0.57	1.33	6.89
AS: 2x PS-PO	0.48	0.49	0.76	0.79	1.27	0.98	1.64	2.77	6.41	0.71	1.39	1.60	1.71	2.30	2.96	6.80	11.52	25.20
AS: 1x PS-PO	11.48	12.42	14.36	14.18	17.62	15.89	19.53	23.52	32.27	12.04	17.00	19.24	20.09	23.50	24.21	33.20	35.93	35.89

AS Intact	87.2 5	86.2 7	84.11	84.27	80.02	82.0 8	77.72	72.10	58.81	86.1 5	80.2 9	77.67	76.70	71.96	69.9 4	55.35	45.19	20.81
RNA-Lipid Adduct Formation																		
SS: 2x Lipid Adducts	0.0	0.0	0.0	0.0	0.0	0.0	0.0	0.0	0.0	0.0	0.0	0.0	0.0	0.1	0.0	0.0	0.1	0.9
SS: 1x Lipid Adducts	0.0	0.1	0.1	0.1	0.2	0.2	0.2	0.2	0.3	0.0	0.2	0.4	0.5	1.7	1.3	1.9	4.0	10.9
SS: Non-lipidated	100. 0	99.9	99.9	99.9	99.8	99.7	99.8	99.7	99.7	100. 0	99.8	99.6	99.5	98.3	98.7	98.0	95.9	88.2
AS: 2x Lipid Adducts	0.0	0.0	0.0	0.0	0.1	0.1	0.1	0.1	0.1	0.0	0.0	0.0	0.1	0.2	0.2	0.2	0.6	2.4
AS: 1x Lipid Adducts	0.0	0.2	0.3	0.4	0.5	0.6	0.5	0.8	0.9	0.1	0.4	0.6	0.7	1.8	2.2	3.0	5.7	13.2
AS: Non-lipidated	100. 0	99.8	99.6	99.6	99.4	99.4	99.4	99.0	99.0	99.9	99.5	99.4	99.3	98.1	97.6	96.8	93.7	84.4

Supplementary Table 3. LC/MS analysis of DOTAP lipid and degradants after siHPRT-LNP storage over four weeks at 25 or 40 °C in two different buffers: (i) phosphate-buffered saline (PBS) 1X, pH 7.4 (“PBS”) or (ii) histidine (10 mM), pH 6.0 (“His”). Selected data from this table is presented in Supplementary Figure 9.

	Initial Time (T0)		4W at 25 °C						4W at 40 °C					
DOTAP	PBS	His	PBS	PBS	PBS	His	His	His	PBS	PBS	PBS	His	His	His
Degradations	Control	Control	Control	Metals	Peroxides	Control	Metals	Peroxides	Control	Metals	Peroxides	Control	Metals	Peroxides
Head Oxidation	0	0	0.03	0.07	0.1	0.02	0.01	0.01	0.18	1.28	0.28	0.05	0.07	0.08
Loss of Oleate + Dienone	0	0	0.04	0.08	0.36	0	0	0	0.1	0.05	0.39	0.02	0.01	0.01
Loss of Oleate + Mono Oxidation	0	0	0.01	0.02	0.08	0	0	0	0.04	0.04	0.15	0.03	0.01	0.01
Loss of Oleate	1.34	1.07	16.59	16.84	16.96	2.86	2.83	5.92	40.58	38.25	41.77	9.12	12.49	14.66
Double Oxidation	0.01	0	0	0.01	0.01	0	0.01	0.02	0.01	0	0	0.01	0.01	0.02
Mono Oxidation at Tail (Dienone)	0.1	0.05	0.1	0.09	0.26	0.05	0.06	0.15	0.08	0.13	0.21	0.06	0.16	0.17
Mono Oxidation at Tail (+O)	0.01	0.01	0.02	0.02	0.03	0.04	0.04	0.11	0.01	0.02	0.02	0.04	0.16	0.16
Double Oxidation at Tail (+2O)	0	0	0.04	0.01	0.15	0	0.01	0.01	0.06	0.05	0.06	0.02	0.04	0.03
Intact	98.54	98.86	83.16	82.86	82.05	97.01	97.04	93.8	58.93	60.18	57.12	90.65	87.05	84.87

Supplementary Table 4. LC/MS analysis of DODMA lipid and degradants after siHPRT-LNP storage over four weeks at 25 or 40 °C in two different buffers: (i) phosphate-buffered saline (PBS) 1X, pH 7.4 (“PBS”) or (ii) histidine (10 mM), pH 6.0 (“His”). Selected data from this table is presented in Supplementary Figure 10.

	Initial Time (T0)		4W at 25 °C						4W at 40 °C					
DODMA	PBS	His	PBS	PBS	PBS	His	His	His	PBS	PBS	PBS	His	His	His
Degradation	Control	Control	Control	Metals	Peroxides	Control	Metals	Peroxides	Control	Metals	Peroxides	Control	Metals	Peroxides
Oxidation and loss of OC18H36	0	0	0	0	0	0	0.01	0	0	0	0.01	0	0	0
Oxidation and loss of C18H36	0.06	0.06	0.14	0.29	0.28	0.29	0.13	0.28	0.28	0.26	0.12	0.28	0.12	0.28
Oxidation and loss of C19H38	0	0	0	0	0	0	0	0	0	0	0	0	0	0
Loss of C19H38	0.29	0.26	0.54	1.05	1.06	1.08	0.5	1.02	1.05	0.93	0.47	1.04	0.48	1.03
Loss of OC18H34	0	0	0	0	0	0	0	0	0	0	0	0	0	0
Loss of C18H34	0.51	0.28	1.23	2.36	2.29	2.65	1.26	2.63	2.37	2.38	1.32	3.39	1.62	3.39
Double Oxidation (+2O-2H)	0	0	0	0.02	0.01	0	0	0	0.01	0.03	0	0	0	0
Double Oxidation (+2O)	0	0	0	0.03	0.02	0.01	0	0.01	0.02	0.05	0.03	0.01	0.01	0.02
Mono Oxidation (O-2H)	0.04	0.04	0.04	0.32	0.25	0.09	0.04	0.08	0.19	0.37	0.2	0.09	0.05	0.11
Mono Oxidation at Tail 1 (O)	0.05	0.06	0.07	0.12	0.13	0.14	0.08	0.15	0.13	0.15	0.09	0.15	0.1	0.17
Mono Oxidation at Tail 2 (O)	0.05	0.05	0.08	0.16	0.15	0.11	0.06	0.12	0.14	0.18	0.1	0.12	0.07	0.12
Head Group Oxidation	0.51	0.5	0.55	0.47	0.54	0.43	0.44	0.53	0.58	0.53	0.73	0.38	0.37	0.54
Head Group Oxidation and loss of CH_2	0.21	0.2	0.44	3.48	1.68	0.61	0.36	0.64	2.62	6.25	2.05	0.89	0.64	1.06
Intact	98.26	98.55	96.9	91.69	93.58	94.58	97.12	94.52	92.58	88.85	94.86	93.63	96.54	93.29

Supplementary Table 5. LC/MS analysis of the major DLin-KC2-DMA lipid variant (-28 Da) and degradants after siHPRT-LNP storage over four weeks at 25 or 40 °C in two different buffers: (i) phosphate-buffered saline (PBS) 1X, pH 7.4 ("PBS") or (ii) histidine (10 mM), pH 6.0 ("His"). Selected data from this table is presented in Supplementary Figure 11.

	Initial Time (T0)		4W at 25 °C						4W at 40 °C					
	PBS	His	PBS	PBS	PBS	His	His	His	PBS	PBS	PBS	His	His	His
Degradations	Control	Control	Control	Metals	Peroxides	Control	Metals	Peroxides	Control	Metals	Peroxides	Control	Metals	Peroxides
Cleavage: C24H36NO	0.02	0.02	0.01	0.17	0.02	0.01	0.02	0.02	0.01	0.22	0.02	0.01	0.02	0.02
Cleavage: C30H55NO4	0	0	0.08	1.47	0.08	0	0.06	0	0.09	2.76	0.13	0	0.16	0
Cleavage: C31H57NO4	0.01	0	0.37	4.51	0.37	0	0.44	0.01	0.35	6.23	0.45	0.01	0.83	0.02
Cleavage: C31H59NO2	0.16	0.18	0.11	0.3	0.1	0.16	0.07	0.17	0.1	0.25	0.11	0.14	0.09	0.14
Cleavage: C32H57NO5	0	0	0.11	2.12	0.11	0	0.94	0	0.06	2	0.08	0	0.6	0
Cleavage: C32H59NO4	0.04	0.02	1.43	10.92	1.41	0.02	2.28	0.03	1.04	11.42	1.3	0.03	2.93	0.05
Cleavage: C33H49NO4	0.01	0.01	0.12	0.67	0.1	0.08	0.09	0.08	0.49	5.74	0.55	0.3	0.38	0.34
Cleavage: C33H59NO3	0.01	0.01	0.25	0.06	0.24	0.01	0.02	0.01	0.29	0.08	0.35	0	0.06	0
Cleavage: C34H61NO3	0.06	0.04	0.38	0.23	0.34	0.03	0.47	0.04	0.16	0.16	0.18	0.03	0.23	0.05
Cleavage: C34H63NO3	0.01	0.01	0.09	0.29	0.08	0.01	0.21	0.02	0.05	0.18	0.06	0.01	0.14	0.02
Cleavage: C35H63NO3	0.01	0.01	0.1	0.24	0.1	0.02	0.05	0.02	0.18	0.08	0.21	0.02	0.31	0.02
Triple Oxidation (3O)	0.06	0.02	0.89	5.73	0.85	0.02	3.63	0.05	0.54	5.02	0.67	0.03	2.48	0.08
Triple Oxidation (3O-2H)	0.05	0.02	1.07	6.74	1.02	0.01	4.07	0.03	0.48	5.91	0.6	0.02	3.06	0.06
Triple Oxidation (3O-4H)	0.02	0	0.62	2.5	0.59	0.01	1.72	0.01	0.27	2.03	0.34	0.01	1.24	0.03
Double Oxidation (2O)	0.73	0.31	5.6	15.83	5.45	0.3	10.18	0.57	3.59	12.47	4.25	0.44	8.17	0.78
Double Oxidation (2O-2H)	0.57	0.27	7.79	12.13	7.54	0.19	9.65	0.28	4.76	10.46	5.49	0.29	8.71	0.51
Double Oxidation (2O-4H)	0.09	0.05	0.85	1.18	0.8	0.07	2.5	0.1	0.48	1.09	0.57	0.12	2.01	0.19
Mono Oxidation (O at Tail 1)	1.65	1.18	6.33	1.09	5.78	1.53	7.83	2.97	4.66	1.19	5.18	2.14	6.48	4.21
Mono Oxidation (O at Tail 2)	0.94	0.81	4.15	7.88	4.08	0.74	6.28	0.88	3.18	5.65	3.3	0.67	6.47	0.85
Head Group Oxidation	0.67	0.3	3.23	5.76	3.31	0.44	6.65	0.91	3.15	8.96	3.68	0.71	8.86	1.62
Oxidation (O-2H)	3.55	2.77	13.79	4.23	13.67	2.6	14.11	3.38	9.78	3.65	10.55	2.41	12.17	3.35
Oxidation and loss of CH2	0.6	0.54	1.76	0.48	1.48	0.58	0.4	0.59	2.17	0.65	2.1	0.76	2.14	0.99
Intact	90.75	93.43	50.85	15.46	52.46	93.17	28.36	89.85	64.12	13.81	59.83	91.85	32.46	86.65

Supplementary Table 6. LC/MS analysis of MC3 lipid and degradants after siHPRT-LNP storage over four weeks at 25 or 40 °C in three different buffers: (i) histidine (10 mM), pH 6.0, (ii) phosphate-buffered saline (PBS) 1X, pH 7.1, and (iii) tris(hydroxymethyl)aminomethane (tris) (10 mM), pH 6.3. Selected data from this table is presented in Figure 4B, 4C. Exemplary structures of each degradant can be found in Supplementary Figure 19.

		siHPRT-LNPs in 10 mM Histidine, 140 mM NaCl, pH 6.0							siHPRT-LNPs in PBS 1X, pH 7.4							siHPRT-LNPs in 10 mM Tris, pH 6.0						
	Mono mass (Da)	T0	25 °C, 2W	25 °C, 4W	40 °C, 3D	40 °C, 10D	40 °C, 2W	40 °C, 4W	T0	25 °C, 2W	25 °C, 4W	40 °C, 3D	40 °C, 10D	40 °C, 2W	40 °C, 4W	T0	25 °C, 2W	25 °C, 4W	40 °C, 3D	40 °C, 10D	40 °C, 2W	40 °C, 4W
Head group (cleaved at ester)	131.0 941	0	0	0	0	0	0	0	0	0	0	0	0	0	0	0	0	0	0	0	0	0
C33H61NO4_ Cle	535.4 595	0	0	0	0	0	0.06	0	0	0	0	0	0.38	0.58	0.51	0	0	0	0	0.01	0.03	0.6
C34H63NO4_ Cle	549.4 752	0	0	0	0	0.01	0.23	0	0.01	0.01	0.03	0.03	1.34	1.89	1.89	0.01	0.01	0.03	0.01	0.08	0.17	2.19
+3O, Triple Oxidation, all at Tail	689.5 953	0	0	0	0	0	0.04	0	0	0.01	0.02	0.03	0.38	0.45	0.36	0	0.01	0.03	0.01	0.05	0.1	0.57
+3O-2H, Triple Oxidation	687.5 796	0	0	0	0	0	0.05	0	0	0.01	0.01	0.02	0.62	0.69	0.51	0	0.01	0.02	0.01	0.05	0.12	0.94
+3O-2H: Triple Oxidation, one at head, two at tail	687.5 796	0	0	0	0	0	0	0	0	0	0	0	0.06	0.07	0.05	0	0	0	0	0	0	0.17
+3O-4H, Triple Oxidation	685.5 645	0	0	0	0	0	0.03	0	0	0	0	0	0.26	0.32	0.24	0	0	0.01	0	0.02	0.04	0.44
+3O_2H, Triple Oxidation	691.6 109	0	0	0	0	0.01	0.1	0.01	0	0.02	0.03	0.03	0.68	0.8	0.78	0	0.01	0.05	0.02	0.1	0.21	1.45
+3O, Triple Oxidation, one at head, two at tail	689.5 953	0.01	0	0.01	0.01	0.01	0.03	0.01	0.03	0.03	0.05	0.05	0.14	0.14	0.13	0.04	0.06	0.06	0.06	0.08	0.12	0.22
+2O-2H, Double Oxidation, one at head, one at tail	671.5 847	0	0	0	0	0	0.02	0	0	0	0	0	0.1	0.17	0.18	0	0	0	0	0	0	0.34
+2O-2H, Double oxidation all at Tail Position 1	671.5 847	0	0	0	0	0	0.04	0	0	0.01	0.02	0.02	0.29	0.36	0.32	0	0.01	0.02	0.01	0.04	0.09	0.44
+2O-2H, Double oxidation all at Tail Position 2	671.5 847	0.01	0	0.02	0.01	0.03	0.49	0.02	0.06	0.19	0.32	0.42	4.42	4.47	3.26	0.07	0.15	0.3	0.2	0.68	1.27	4.49
+2O-4H, Double Oxidation, all at Tail	669.5 69	0	0	0	0	0	0.02	0	0	0	0.01	0	0.22	0.29	0.29	0	0	0.01	0	0.02	0.05	0.44
+2O, Double Oxidation, one at head, one at tail	673.6 004	0.06	0.02	0.04	0.03	0.08	0.1	0.05	0.1	0.09	0.13	0.12	0.26	0.23	0.25	0.11	0.2	0.17	0.18	0.25	0.37	0.39
+2O, Double Oxidation, all at Tail Position 1	673.6 004	0	0	0	0	0	0.02	0	0	0	0.01	0.01	0.12	0.15	0.15	0	0	0.01	0	0.02	0.04	0.22
+2O, Double Oxidation, all at Tail Position 2	673.6 004	0	0	0.02	0.02	0.03	0.43	0.02	0.04	0.13	0.26	0.2	3.18	3.62	2.89	0.04	0.11	0.26	0.12	0.41	0.76	3.63
+O-2H, Mono Oxidation, at Tail	655.5 898	0.03	0.02	0.07	0.05	0.14	0.89	0.12	0.07	0.3	0.74	0.44	6.52	6.91	6.9	0.08	0.22	0.61	0.26	1.16	2.33	8.05
+O, Mono Oxidation, at Tail Position 1	657.6 054	0.01	0.02	0.11	0.04	0.2	0.54	0.21	0.03	0.19	0.39	0.21	2.94	3.44	3.59	0.03	0.14	0.38	0.13	0.65	1.21	4.12
+O, Mono Oxidation, at Tail Position 2	657.6 054	0	0	0	0	0.01	0.08	0.01	0	0.01	0.02	0.02	0.59	0.77	0.68	0.01	0.01	0.02	0.02	0.06	0.14	0.83
+O, Mono Oxidation, at Head	657.6 054	0.02	0.05	0.07	0.03	0.06	0.16	0.13	0.04	0.12	0.25	0.12	1.85	2.21	2.58	0.02	0.09	0.23	0.07	0.37	0.7	3.26
CH2_Oxidation , Oxidation and lost Methyl at Head	627.5 949	0.03	0.03	0.06	0.03	0.04	0.14	0.07	0.05	0.18	0.34	0.4	1.6	1.46	0.88	0.07	0.13	0.34	0.19	0.7	1.31	2.14
Intact MC3	641.6 105	99.83	99.85	99.6	99.76	99.38	96.52	99.3 6	99.57	98.7	97.34	97.87	74.07	70.97	73.57	99.52	98.84	97.46	98.69	95.24	90.95	65.06

Supplementary Table 7. LC/MS analysis of siHPRT, degradants, and siHPRT-lipid adduct formation after siHPRT-LNP storage over four weeks at 25 or 40 °C in three different buffers: (i) histidine (10 mM), pH 6.0, (ii) phosphate-buffered saline (PBS) 1X, pH 7.1, and (iii) tris(hydroxymethyl)aminomethane (tris) (10 mM), pH 6.3. Selected data from this table is presented in Figure 4E, 4F. Legend: SS: sense strand; AS: antisense strand. First values represent the number of nucleosides present in the byproduct and where cleavage occurred (*e.g.,* “SS: 1-19” indicates that the last two nucleotides were cleaved, while “SS: 2-21” indicates that the first nucleotide was cleaved). Unless otherwise described, degradants result in a 5’-OH. In other instances, the phosphate is retained at the 5’ position and is denoted with a “P” (*e.g.,* “P-2-21”). Number of phosphorothioate-to-phosphodiester transformations are denoted by “#x PS-PO.” Defluorination of 2’-fluoro-deoxyribonucleosides is denoted as “#x loss F.” The number of RNA-lipid adducts (reactivity between **1** and **2**, as described in Figure 4) is denoted by “#x Lipid Adducts.”

	siHPRT-LNPs in 10 mM Histidine, 140 mM NaCl, pH 6.0							siHPRT-LNPs in PBS 1X, pH 7.4							siHPRT-LNPs in 10 mM Tris, pH 6.3						
siRNA Degradation	T0	25 °C, 2W	25 °C, 4W	40 °C, 3D	40 °C, 10D	40 °C, 2W	40 °C, 4W	T0	25 °C, 2W	25 °C, 4W	40 °C, 3D	40 °C, 10D	40 °C, 2W	40 °C, 4W	T0	25 °C, 2W	25 °C, 4W	40 °C, 3D	40 °C, 10D	40 °C, 2W	40 °C, 4W
AS: 2-23, 3x PS-PO	0	0	0	0	0	0	0	0	0	0	0	0.1	0.3	0.5	0	0	0	0	0	0.1	1.5
AS: 2-23, 1x PS-PO	0.3	0.4	0.2	0.2	0.3	0.3	0.5	0.2	0.2	0.1	0.2	0.1	0.3	0.4	0.1	0.1	0.3	0.2	0.2	0.2	0
AS: 2-23, 2x PS-PO	0	0	0	0	0	0	0.1	0	0	0	0	0.2	0.7	0.9	0	0	0	0	0.2	0.3	0.4
AS: 1-21, 2x PS-PO	0	0	0	0	0	0	0	0	0	0	0	0.9	1.7	2	0	0	0	0	0.1	0.5	10.5
AS: 1-21, 2x PS-PO, 1x loss F	0	0	0	0	0	0	0	0	0	0	0	0.3	1.4	1.8	0	0	0	0	0	0.1	0.5
AS: 1-21, 1x PS-PO	0.5	0.6	0	0	0	0.1	0.1	0	0.2	0.5	0.2	1.8	2.1	2.1	0	0.1	0.4	0.1	0.9	1.9	6
AS: 1-21, PS-PO, 1x loss F	0.2	0.2	0.1	0	0.1	0.1	0.2	0	0.1	0.1	0.1	0.4	1.4	1.5	0	0	0	0	0.1	0.1	0.6
AS: 4x PS-PO	0	0	0	0	0	0	0	0	0	0	0	0.3	1.4	1.8	0	0	0	0	0	0.1	8.4
AS: 3x PS-PO	0.1	0	0	0	0	0.2	0	0	0	0.1	0.1	7	10.1	12.5	0	0	0	0	0.6	2.1	35.7
AS: 2x PS-PO	0.2	0.1	0.2	0.2	0.3	0.9	0.7	0	1.1	3.6	1.9	28	29.5	31.9	0.1	0.3	1.7	0.5	8	17	28.9
AS: 1x PS-PO	3.3	5.6	7	5.1	7.7	9.4	13.7	3.4	17.9	29.7	21.6	39	35.4	33.2	2.1	7.5	22.7	9.9	40.1	46.9	6.5
AS Intact	95.4	93	92.5	94.5	91.7	89.1	84.8	96.4	80.4	65.9	75.9	21.7	15.8	11.5	97.8	92	74.9	89.3	49.8	30.8	0.9
SS: P-3-21	0	0	0	0	0	0	0	0	0	0	0	0.4	1	1.4	0	0	0	0	0	0	0.1
SS: P-3-21, PS-PO	0	0	0	0	0	0	0	0	0	0	0	0.2	0.5	0.9	0	0	0	0	0	0	0.2
SS3-21	0.1	0.1	0	0	0	0.1	0	0	0.1	0.1	0.1	0.2	0.4	0.5	0	0	0	0	0	0	0.2
SS: P-2-21	0	0	0	0	0	0	0	0	0	0.1	0	0.5	1	1.1	0	0	0	0	0.1	0.1	0.2
SS: P-2-21, 1x PS-PO	0	0	0	0	0	0	0	0	0	0	0	0.2	0.7	1.1	0	0	0	0	0	0	1
SS: 2-21	0.4	0.4	0.4	0.3	0.4	0.4	0.4	0.3	0.7	0.8	0.7	2.7	3.5	3.3	0.2	0.3	0.5	0.3	1	1.6	2.4
SS: 2-21, 2x PS-PO	0	0	0	0	0	0	0	0	0	0	0	0.1	0.5	0.8	0	0	0	0	0	0	3.8
SS: 2-21, 1x PS-PO	0	0	0	0	0	0.1	0	0	0	0	0	0.9	2.4	3.1	0	0	0	0	0.1	0.4	5.6
SS: 1-19	0	0	0	0	0	0	0	0	0	0	0	0.2	0.5	0.6	0	0	0	0	0.1	0.1	0.6
SS: 1-19, 1x PS-PO	0	0	0	0	0	0	0	0	0	0	0	0	0	0.1	0	0	0	0	0	0	0.2

SS: 3x PS-PO	0	0	0	0	0	0.1	0	0	0	0	0	0.9	2.9	4.6	0	0	0	0	0.1	0.1	14.5
SS: 2x PS-PO	0.4	0.2	0.1	0	0.1	0.3	0.1	0	0	0.3	0.4	9.8	15.9	19	0	0	0.2	0.1	1	2.8	32.5
SS: 1x PS-PO	2.4	4.5	3.4	2.6	3.7	5.1	6	1.7	7	12.2	9.8	38.2	38.4	37.7	1.1	2.9	7.7	4.1	19.7	30.5	34.9
SS Intact	96.7	94.8	96.1	97	95.8	93.8	93.3	98	92.1	86.5	88.9	45.8	32.3	25.7	98.6	96.7	91.5	95.4	78.1	64.4	3.9
RNA-Lipid Adduct Formation																					
AS Lipid Modification	0.58	0.06	0.08	0.07	0.06	0.44	0.14	0.39	2.52	5.63	4.72	14.6	18.2	12.64	1.04	3.21	10.03	4.57	22.4	34.31	67.61
AS: Non-lipidated	99.42	99.94	99.92	99.93	99.94	99.56	99.86	99.61	97.48	94.37	95.28	85.4	81.8	87.36	98.96	96.79	89.97	95.43	77.6	65.69	32.39
SS: Lipid modification	0.28	0.05	0.07	0.07	0.08	1.5	0.12	0.42	1.52	3.41	2.62	28.87	34.87	24.58	0.93	2.64	6.75	3.16	12.75	24.95	59.78
SS: Non-lipidated	99.72	99.95	99.93	99.93	99.92	98.5	99.88	99.58	98.48	96.59	97.38	71.13	65.13	75.42	99.07	97.36	93.25	96.84	87.25	75.05	40.22

Supplementary Table 8. Comparing the stability of three different ionizable lipids (MC3 (**1**), ALC-0315, and SM-102) stabilizing EPO mRNA-LNPs within phosphate and histidine-containing buffers. RNA-LNP solutions were stored at either 2–8 °C or room temperature (22–25 °C).

Intact Ionizable Lipid	EPO mRNA-LNPs in PBS 1X, pH 7.4					EPO mRNA-LNPs in 10 mM Histidine + 140 mM NaCl, pH 6				
	T0	2–8 °C, 2w	2–8 °C, 4w	RT, 2w	RT, 4w	T0	2–8 °C, 2w	2–8 °C, 4w	RT, 2w	RT, 4w
MC3 (**1**) Intact	93.91	91.54	88.87	62.13	57.78	95.74	95.72	95.66	95.82	95.86
ALC-0315 Intact	99.94	99.93	99.93	99.94	99.94	99.9	99.91	99.93	99.92	99.9
SM-102 Intact	99.68	99.68	99.61	99.62	99.76	99.68	99.75	99.69	99.51	99.69
