# Supplementary material for: Buffer optimization of siRNA-lipid nanoparticles mitigates lipid oxidation and RNA-lipid adduct formation
Source: Nat Commun. 2025 Sep 25;16:8380. doi: 10.1038/s41467-025-63651-4 (PMC12462517; doi:10.1038/s41467-025-63651-4)
Supplement: Supplementary file 2 — Description of Additional Supplementary Files [file 41467_2025_63651_MOESM2_ESM.pdf]

Description of Additional Supplementary Files

Supplementary Data 1. Optimized Cartesian coordinates (Å) of Compound A within Supplementary Figure 13.

Supplementary Data 2. Optimized Cartesian coordinates (Å) of Compound B within Supplementary Figure 13.

Supplementary Data 3. Optimized Cartesian coordinates (Å) of Compound C within Supplementary Figure 13.

Supplementary Data 4. Optimized Cartesian coordinates (Å) of Compound D within Supplementary Figure 13.

Supplementary Data 5. Optimized Cartesian coordinates (Å) of Compound E within Supplementary Figure 13.

Supplementary Data 6. Optimized Cartesian coordinates (Å) of Compound F within Supplementary Figure 13.

Supplementary Data 7. Optimized Cartesian coordinates (Å) of Compound G within Supplementary Figure 13.

Supplementary Data 8. Optimized Cartesian coordinates (Å) of Compound H within Supplementary Figure 13.
